# Identifying Prognostic Biomarkers and Key Pathways in Renal Clear Cell Carcinoma: A Pilot Study Using Integrated miRNA and Gene Expression Analysis

**DOI:** 10.1155/bri/3213941

**Published:** 2026-02-26

**Authors:** Hamed Manoochehri, Kosar Mirzaee, Amir Taherkhani, Mahmoud Gholyaf

**Affiliations:** ^1^ The Persian Gulf Marine Biotechnology Research Center, The Persian Gulf Biomedical Sciences Research Institute, Bushehr University of Medical Sciences, Bushehr, Iran, bpums.ac.ir; ^2^ Department of Internal Medicine, School of Medicine, Hamadan University of Medical Sciences, Hamadan, Iran, umsha.ac.ir; ^3^ Urology and Nephrology Research Center, Avicenna Institute of Clinical Sciences, Hamadan University of Medical Sciences, Hamadan, Iran, umsha.ac.ir; ^4^ Research Center for Molecular Medicine, Institute of Cancer, Hamadan University of Medical Sciences, Hamadan, Iran, umsha.ac.ir

**Keywords:** bioinformatics, clear cell renal cell carcinoma, hub genes, miRNA, prognostic biomarkers, protein interaction networks

## Abstract

**Background and Objective:**

Renal clear cell carcinoma (RCCC) stands out as a prevalent and aggressive subtype of kidney cancer characterized by a challenging prognosis. The need to enhance patient outcomes in RCCC underscores the significance of identifying prognostic biomarkers and therapeutic targets. MicroRNAs (miRNAs) and the signaling pathways orchestrating RCCC pathogenesis emerge as promising candidates for such endeavors.

**Methods:**

This study utilized publicly available gene expression data to compare miRNA profiles in nine RCCC and 11 normal kidney tissues. Rigorous bioinformatics analyses were employed to identify differentially expressed miRNAs and their associated gene targets. Prognostic significance was assessed, and a protein–protein interaction network was constructed to highlight pivotal RCCC hub genes. The expression and prognostic value of key hub genes and miRNAs were further validated in independent cohorts, including the GEO dataset GSE76351 and the TCGA–KIRC cohort via the Kaplan–Meier plotter. Expression of RUNX2 was confirmed using real‐time PCR in five cancer and five normal renal tissues.

**Results:**

Fifteen DEMs were identified alongside 74 hub genes. The downregulation of miR‐26a‐1‐3p, miR‐144–3p, and miR‐144–5p was associated with a poorer prognosis in RCCC. The overexpression of CDK1 and RUNX2 was validated in an independent GEO dataset and correlated with decreased patient survival in the TCGA–KIRC cohort. Furthermore, a statistically significant but modest inverse association was observed between miR‐26a‐1‐3p and RUNX2 expression, indicating a possible miRNA–mRNA relationship. Significant enrichment was observed in pathways related to PI3K–Akt, MAPK, apoptosis, and cell cycle. The overexpression of RUNX2 was confirmed in our patient samples (*p* value< 0.05).

**Conclusion:**

This multistep validation study confirms that specific miRNAs and hub genes, particularly the miR‐26a‐1‐3p/RUNX2 axis, are potential prognostic indicators in RCCC. A comprehensive understanding of these biomarkers and their enriched signaling pathways provides deeper insight into the molecular underpinnings of RCCC, uncovering potential therapeutic opportunities.

## 1. Introduction

Kidney cancer, constituting approximately 4.2% of all malignancies, significantly impacts human health and well‐being [1]. Renal clear cell carcinoma (RCCC) accounts for about 85% of all renal cell carcinoma (RCC) cases [2, 3]. This prevalent form of cancer exhibits a high incidence rate, elevated mortality, and suboptimal response to conventional treatments. Originating in the epithelial cells of the proximal convoluted tubule, RCCC exhibits a complex molecular landscape characterized by copy‐number alterations, including loss of 3 p and inactivation of the von Hippel–Lindau (VHL) tumor suppressor gene. These genetic anomalies disrupt metabolic and immune‐response pathways, contributing to the initiation and progression of the disease [4].

The prognostic landscape of RCCC involves various genetic and molecular markers. SETD2, PBRM1, and BAP1 mutations promote genomic instability and metastatic potential [4]. Ongoing research endeavors focus on identifying mutations, gene expression, and proteomic signatures as potential biomarkers for early diagnosis and prognostication [4]. Recognizing the significance of targeted therapy, previous reports underscore its potential to reverse the poor prognosis associated with RCCC [5–7].

Efforts are underway to develop an efficient prognostic model to accurately predict outcomes and therapeutic benefits for RCCC patients [8]. While targeted treatment, chemotherapy, and immunotherapy are preferred modalities for advanced clear cell carcinoma [9], their therapeutic outcomes are hindered by factors such as the lack of consideration of individual variation, the absence of reliable prognostic biomarkers, and medication resistance [9]. Consequently, a critical imperative exists to identify accurate predictive biomarkers to enhance prognostic precision and overall therapeutic efficacy for patients with kidney renal clear cell carcinoma (KIRC) [10].

MicroRNAs (miRNAs) are short, noncoding RNAs (20–23 nucleotides) that control gene transcription. These small RNA molecules bind to complementary sequences in the 3′‐UTRs of their target genes, leading to mRNA degradation or translation inhibition [11]. Displaying tissue‐specific expression [12], dysregulation of miRNAs is linked to tumorigenesis, presenting them as promising targets for cancer therapy. In cancer treatment, miRNAs can act as either oncogenes or tumor suppressors, providing a novel therapeutic avenue [13]. Scientists are increasingly exploring miRNAs as potential cancer biomarkers, using extensive datasets from gene and miRNA expression microarrays to discover biomarkers for diagnosis, prognosis, and therapeutic strategies [12, 14].

The principal aim of this investigation was to employ cutting‐edge, robust methodologies to identify differentially expressed miRNAs (DEMs) in RCCC tissues compared with adjacent normal kidney samples. The central focus of the study was to establish a comprehensive set of prognostic markers, encompassing miRNAs and their target genes. These markers were intended to accurately predict the prognosis of RCCC patients and potentially unveil novel drug targets. Additionally, the inquiry analyzed hub genes, cluster genes, signaling pathways, and biological processes (BPs) that significantly influence the malignant transformation from normal kidney tissue to RCCC.

To achieve this objective, advanced techniques were deployed to reanalyze the GSE23085 microarray dataset, initially curated by White et al. [15]. The overarching goal was to provide distinctive insights surpassing the methodologies employed in the initial study, with the ultimate aim of revealing crucial molecular mechanisms underlying RCCC. This pursuit, in turn, holds the potential for substantial advancements in the comprehension and treatment of this disease.

## 2. Materials and Methods

### 2.1. Sample Collection

The research conducted by White et al. [15] examined miRNA expression profiles in RCCC tissues and their corresponding healthy specimens sourced from St. Michael’s Hospital in Toronto, Ontario, Canada. A total of 20 pairs of tissue samples, acquired post‐total laparoscopic tumor nephrectomy, were subjected to microarray analysis and preserved at −80°C. Histological verification of all cases was independently performed by two pathologists, in accordance with the research ethics board’s guidelines and regulations at St. Michael’s Hospital. The microarray data are available in the GEO repository under the identifier GSE23085 [15, 16].

### 2.2. miRNA Dataset Recovery and Statistical Analysis

The dataset GSE23085 included genome‐wide miRNA profiles from RCCC tissue samples (*n* = 9) and matched healthy kidney tissues (*n* = 11), profiled using the GPL10415 platform (LC_MRA‐1001_miRHuman_13.0_090309 miRNA ID version). After retrieving the raw dataset files, quality control checks were performed, resulting in the identification of 724 human miRNAs profiled across 20 tissue samples. For miRNAs corresponding to duplicate probes, average expression values were taken. Differential expression analysis between RCCC and healthy kidney samples was done using the GEO2R analysis tool. The DEMs were defined based on a false discovery rate (FDR) < 0.01 and an absolute log2 fold change (FC) |log2FC| > 1.585, corresponding to raw FC values < 1/3 or > 3. This threshold was selected to analyze miRNAs with the most pronounced differential expression between RCCC and healthy tissue.

### 2.3. Network Analysis Based on RCCC‐Related Genes

The targets of DEMs were identified by searching the miRWalk 2.0 database (https://mirwalk.umm.uni-heidelberg.de/), an archive of predicted and validated miRNA–target interactions [17]. To further filter the predicted interactions, only targets present in miRTarBase (https://mirtarbase.mbc.nctu.edu.tw/php/index.php) [18], a database of experimentally validated miRNA–target interactions with strong evidence scores, were designated as validated targets of the DEMs. To compile a set of genes associated with RCCC reported in previous studies, Version 7.0 of the DisGeNET database (https://www.disgenet.org/) [19] was consulted. The dataset with ID C0007134 related to RCCC was obtained, and genes were extracted. By comparing the DEM target genes and DisGeNET genes using interactive Venn diagrams (https://bioinformatics.psb.ugent.be/webtools/Venn/), a comprehensive set of genes linked to RCCC was identified. Protein interactions between these RCCC‐related genes were visualized using the STRING database (Version 12.0, https://string-db.org/) [20], a resource for protein–protein interaction networks. After removing disconnected nodes [21], the connected network was imported into Cytoscape (Version 3.10.1, https://cytoscape.org/), an open‐source platform for network analysis [22]. Hub proteins were defined as those with a higher‐than‐average degree and betweenness centralities across all nodes. Finally, densely connected modules within the network were detected using the MCODE plugin in Cytoscape [23].

### 2.4. Functional Enrichment Analysis

The gene ontology (GO) and pathway enrichment analyses were performed using the g:Profiler (https://biit.cs.ut.ee/gprofiler/gost) [24]. The g:Profiler integrates functional information from a variety of databases, including GO terms for cellular components (CCs), molecular functions (MFs), and BPs; KEGG, Reactome, and WikiPathways; miRTarBase miRNA targets; tissue specificity data from Human Protein Atlas; CORUM protein complexes; and disease phenotypes from Human Phenotype Ontology. Terms with an FDR under 0.05 and containing at least 10 enriched genes were considered statistically significant.

Gene set enrichment for CCs and MFs was performed using g:Profiler, with the input set equal to the intersection of DEM targets and DisGeNET‐derived RCCC genes. Subsequently, pathway and BP analyses were restricted to the genes located within the primary clusters of the PIM.

### 2.5. Prognostic Impact of the Hub Genes and DEMs

Given the key roles that hub genes play in RCCC, their prognostic potential was evaluated using the Gene Expression Profiling Interactive Analysis 2 (GEPIA2) database (http://gepia2.cancer-pku.cn/) [25]. GEPIA2 performs survival analyses by reanalyzing RNA sequencing expression data from The Cancer Genome Atlas (TCGA) and Genotype‐Tissue Expression (GTEx) typical tissue databases. Genes with Log‐rank test and hazard ratio (HR) *p* values under 0.05 were designated prognostic biomarkers. To provide further insight, the predictive value of gene combinations was also assessed [26]. Moreover, the prognostic significance of DEMs in KIRC was evaluated utilizing the Kaplan–Meier plotter, accessible at https://kmplot.com/analysis/.

In addition, the expression patterns of prognostic DEMs were analyzed using the UALCAN online platform (http://ualcan.path.uab.edu/) [27]. A comparative analysis was performed between renal carcinoma tissues and normal renal samples, with statistical significance defined as a *p* value < 0.05.

### 2.6. Box plot and stage plot analyses

The mRNA expression patterns of genes associated with unfavorable prognosis were examined in 523 KIRC tissue samples and 100 healthy renal tissue samples using the boxplots provided in the GEPIA2 database [25]. Additionally, stage plot analysis was performed to evaluate expression changes in these unfavorable prognostic markers across KIRC Stages I–IV. In stage plot analysis, boxplots depict the distribution of gene expression in each cancer stage, allowing assessment of variability across stages. ANOVA testing determines if the mean expression levels differ significantly between any two stages, with Pr (> F) < 0.05 indicating a significant difference. Implementing stage plot analysis facilitates determining whether biomarker expression tracks with KIRC progression. Both boxplot and stage plot analyses aid in evaluating how the harmful prognostic genes are differentially expressed between malignant and healthy kidney tissues and during stepwise renal carcinoma development.

### 2.7. Real‐Time PCR (RT‐PCR)

Ten specimens (five renal carcinoma tissues and five noncancerous normal renal tissues) were obtained from the Imam Khomeini Hospital Cancer Institute (Tehran, Iran) in compliance with ethical guidelines (IR.UMSHA.REC.1403.352). Total RNA was extracted from 50 mg of homogenized tissue using RNX + reagent (Sinaclon, Iran) following the manufacturer’s protocol. RNA quality and quantity were evaluated via spectrophotometric analysis (NanoDrop, A260/A280 ratio) and 1.5% agarose gel electrophoresis. Complementary DNA (cDNA) synthesis was performed with 1.0 μg of total RNA using the RevertAid First Strand cDNA Synthesis Kit (Thermo Fisher Scientific, USA) in a 20 μL reaction volume.

For quantitative qRT‐PCR, synthesized cDNA, gene‐specific primers (Supporting Data 1), and RealQ Plus 2 × Master Mix Green (Ampliqon, Denmark) were used in a Corbett Research RG‐6000 thermocycler with a reaction volume of 25 μL, prepared according to the Master Mix instructions. The thermal cycling protocol included an initial denaturation at 95°C for 7 min, followed by 40 cycles of denaturation (95°C, 20 s), annealing (58°C–61°C, 20 s), and extension (72°C, 20 s). Postamplification melt curve analysis and 1.5% agarose gel electrophoresis confirmed primer specificity. Relative gene expression levels were analyzed and shown using the −ΔCT measure, with GAPDH as the endogenous reference gene. Group comparisons were carried out using an independent samples *t*‐test, and statistical significance was defined as *p* < 0.05.

### 2.8. Validation of Hub Gene Expression and Prognostic Value in Independent Cohorts

To independently validate the expression and prognostic significance of hub genes, two additional lines of investigation were conducted. First, gene expression data from the GEO dataset GSE76351 (https://www.ncbi.nlm.nih.gov/geo/query/acc.cgi?acc=GSE76351) were analyzed using the GEO2R tool. This dataset comprises gene expression profiles from tumor and normal kidney tissues of patients aged 50–75 years, generated using the Affymetrix Human Gene 1.1 ST Array platform (GPL11532). The expression levels of two of the hub genes were compared between KIRC and healthy control tissues.

Second, to evaluate their prognostic value, the Kaplan–Meier plotter online tool (https://kmplot.com/analysis/) [28] was used. This platform was used to assess the correlation between gene expression (measured by RNA‐Seq) and overall survival in patients with RCCC.

### 2.9. Analysis of miRNA–Target Gene Expression Correlation

To investigate the potential regulatory relationships between the key prognostic miRNAs and the prognostic hub genes, an in silico correlation analysis was performed. This was done using the starBase database (https://rnasysu.com/encori/index.php) [29], across 517 samples from the TCGA–KIRC cohort. Pearson correlation coefficients (*r*) and corresponding *p* values were calculated and recorded to assess the strength and statistical significance of each relationship.

### 2.10. Writing Style and Grammar Review

The manuscript’s writing style was edited by an AI tool, and its grammar was subsequently verified with Grammarly Premium.

## 3. Results

### 3.1. DEMs in RCCC

**Figure 1:**
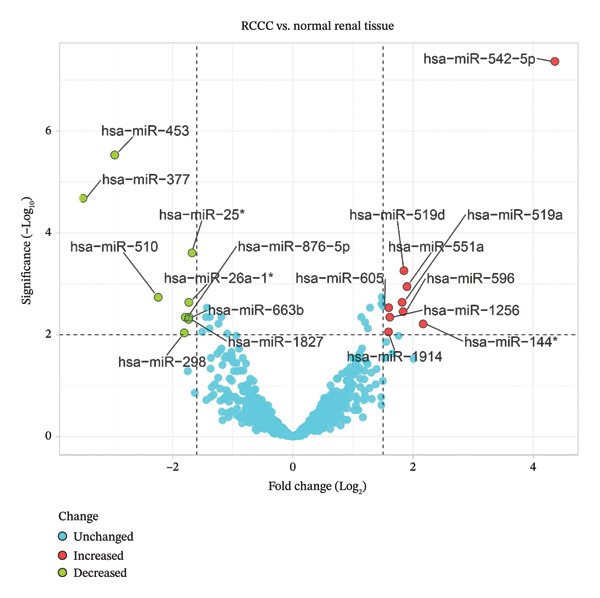
Volcano plot of miRNAs in clear cell renal cell carcinoma compared to the normal renal tissues.

Compared to healthy tissue specimens, 15 DEMs were identified in RCCC tissues using GEO2R analysis. The identification criteria were the FDR < 0.01 and |log2 FC| > 1.585. Of the 15 DEMs, nine were upregulated, and six were downregulated in RCCC relative to healthy tissues (Table 1). Volcano plots visually demonstrating the DEMs between the RCCC and healthy tissue groups based on ‒log10 FDR and log2 FC values were generated using the VolcaNoseR Shiny app, a web‐based tool available at https://huygens.science.uva.nl/VolcaNoseR (Figure 1) [30].

**Table 1:** Differentially expressed miRNAs in clear cell renal cell carcinoma compared with the normal renal tissues were identified by GEO2R.

A, Upregulated			
miRNA	FDR	FC	Log2 FC
hsa‐miR‐542‐5p	4.30E‐08	20.535	4.36
hsa‐miR‐144	6.08E‐03	4.469	2.16
hsa‐miR‐551a	1.13E‐03	3.732	1.9
hsa‐miR‐519d	5.51E‐04	3.605	1.85
hsa‐miR‐596	3.50E‐03	3.555	1.83
hsa‐miR‐519a	2.30E‐03	3.531	1.82
hsa‐miR‐1256	4.50E‐03	3.053	1.61
hsa‐miR‐605	2.94E‐03	3.010	1.59
hsa‐miR‐1914	8.78E‐03	3.010	1.59

**B, Downregulated**			

**miRNA**	**FDR**	**FC**	**Log2 FC**

hsa‐miR‐25	2.45E‐04	0.312	−1.68
hsa‐miR‐26a‐1	2.30E‐03	0.301	−1.73
hsa‐miR‐663b	4.50E‐03	0.301	−1.73
hsa‐miR‐1827	4.95E‐03	0.299	−1.74
hsa‐miR‐876–5p	4.50E‐03	0.289	−1.79
hsa‐miR‐298	9.16E‐03	0.285	−1.81
hsa‐miR‐510	1.83E‐03	0.212	−2.24
hsa‐miR‐377	2.09E‐05	0.089	−3.49

Abbreviations: FC, fold change; FDR, false discovery rate.

### 3.2. Modules, Hub Genes, Pathways, and GO Terms

The miRWalk 2.0 database successfully identified 4495 genes as DEM targets, with experimental validation. Additionally, leveraging the DisGeNET database, a comprehensive set of 2084 genes was identified as associated with RCCC. Notably, an intersection of 424 genes was observed between these two datasets, denoted as RCCC‐related genes (Supporting Data 2). Subsequently, this set of genes was used as input to the STRING database, facilitating the construction of a protein interaction map (PIM). After removing isolated nodes, a connected network comprising 396 proteins and 3704 interactions was imported into Cytoscape 3.10.1 for further analysis. The average degree centrality and betweenness centrality values calculated across the network were 18.7 and 0.004, respectively. Based on these centrality metrics, 74 nodes were designated as hub genes potentially relevant to the etiology of RCCC (Supporting Data 3). Table 2 lists the top 30 high‐degree hub genes in descending order of degree centrality.

**Table 2:** Top‐30 hub genes based on the degree centrality in the PIM associated with RCCC patients.

Name	Degree	Betweenness
TP53	171	0.142
AKT1	170	0.112
MYC	148	0.089
EGFR	143	0.073
SRC	126	0.072
VEGFA	126	0.047
EGF	107	0.025
IL1B	106	0.040
CDH1	99	0.030
ERBB2	97	0.016
FGF2	75	0.009
TLR4	73	0.021
BCL2L1	70	0.008
MDM2	66	0.014
ICAM1	66	0.007
CDC42	64	0.024
MAPK8	64	0.007
IGF1R	64	0.005
PIK3R1	63	0.010
SMAD4	62	0.014
BRCA1	57	0.008
CXCL12	57	0.007
MMP2	57	0.005
CDKN1A	57	0.004
CREB1	56	0.013
SMAD3	56	0.008
RELA	56	0.007
CASP8	56	0.006
TRAF6	50	0.019
NANOG	50	0.019

Abbreviations: PIM, protein interaction map; RCCC, renal clear cell carcinoma.

**Figure 2:**
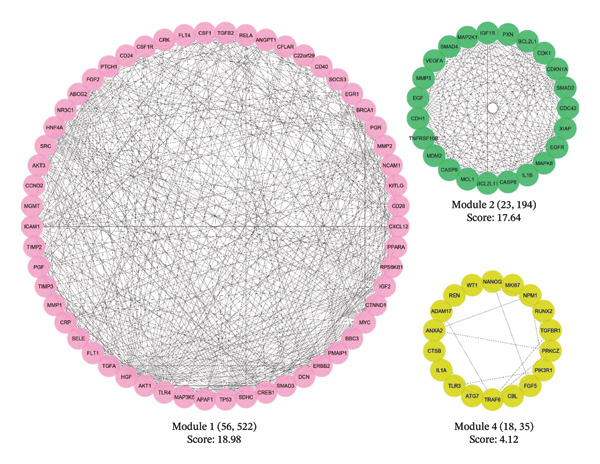
Three significant protein interaction modules (clusters) were identified by the MCODE plugin within the protein interaction map associated with tumorigenesis in RCCC. MCODE, molecular complex detection; RCCC, renal clear cell carcinoma.

Further network analysis was conducted utilizing the MCODE plugin in Cytoscape. This revealed three significant gene subnetworks (designated Cluster 1, Cluster 2, and Cluster 3) participating in pathways and BPs implicated in RCCC pathogenesis. Cluster 1 contained the highest number of 56 genes with 522 connecting edges and demonstrated the maximal MCODE clustering score of 18.98. The three clusters are visualized in Figure 2, which summarizes the respective MCODE scores, number of constituent genes, and interaction edges.

Pathway enrichment analysis revealed “Pathway in cancer” (KEGG:05,200), “PI3K–Akt signaling pathway” (KEGG:04,151), and “MAPK signaling pathway” (KEGG:04,010) as the most significantly dysregulated signaling pathways involved in the malignant transformation of normal renal tissue into RCCC. Additionally, “Regulation of programmed cell death” (GO:0043067), “Regulation of signal transduction” (GO:0009966), and “Regulation of apoptotic process” (GO:0042981) emerged as the top BPs mediating kidney tumorigenesis. Regarding CC categories, “Cytoplasm” (GO:0005737) and “Cell periphery” (GO:0071944) were most substantially impacted in RCCC. Finally, at the MF level, “Protein binding” (GO:0005515) and “Enzyme binding” (GO:0019899) were the most enriched terms. Supporting Data 4–7 present pathway names and GO annotations showing statistical enrichment during the malignant transformation of kidney tissue into a cancerous state. The top‐10 enriched terms from this analysis are highlighted in Figure 3.

Figure 3The most significantly enriched (a) pathways, (b) biological processes, (c) molecular functions, and (d) cellular components in RCCC are presented. The *x*‐axis displays the name of the pathway or GO term. The *y*‐axis shows the negative Log10 of the FDR value for each term, indicating the statistical significance of its enrichment in RCCC. Higher Log10 (FDR) values indicate greater enrichment for the disease state. GO, gene ontology; FDR, false discovery rate; RCCC, renal clear cell carcinoma.

**Figure:**
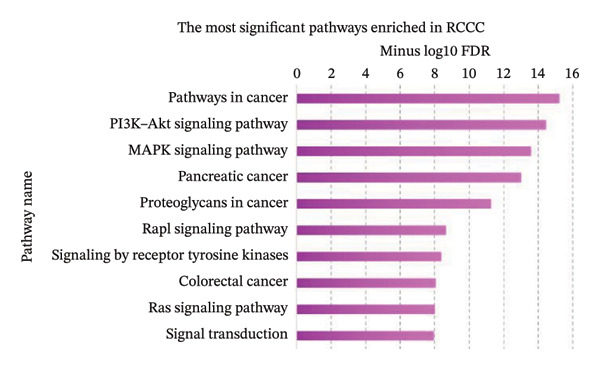
(a)

**Figure:**
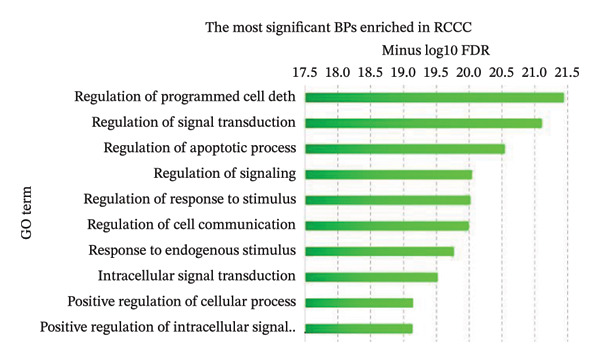
(b)

**Figure:**
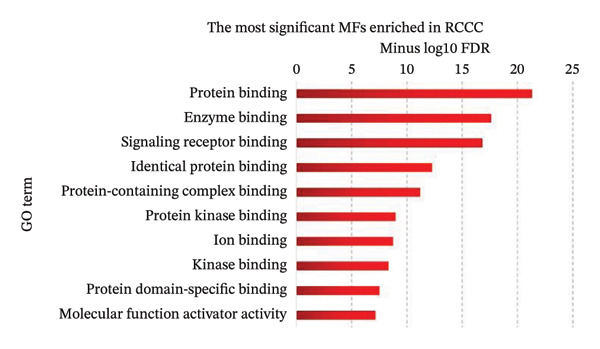
(c)

**Figure:**
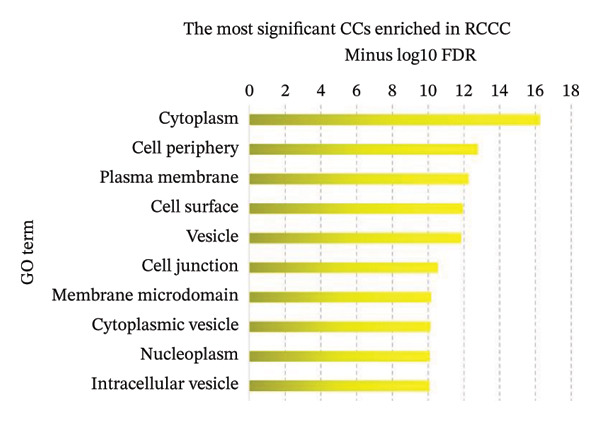
(d)

### 3.3. Prognostic Markers

The GEPIA2 tool identified significant correlations between the overexpression of CRP, CDK1, and RUNX2 and an unfavorable prognosis in RCCC, indicating that they are negative prognostic markers. CRP had the most significant prognostic impact, with an HR of 1.8 and a Log‐rank *p* value of 3.40E‐04. Conversely, 38 genes were identified as positive markers in RCCC, with ERBB2 showing the most significant outcome, with an HR of 0.39 and a Log‐rank *p* value of 5.50E‐09. The combination of CRP, CDK1, and RUNX2 in a gene signature yielded an HR of 1.7 and a Log‐rank *p* value of 0.0012. Table 3 comprehensively lists negative and positive markers in RCCC patients. Additionally, Supporting Data 8 presents the Kaplan–Meier curves for negative markers, along with their associated signatures.

**Table 3:** Forty‐one hub genes in the RCCC–PIM were prognostic for the disease.

	**A, Single gene**			
	**Gene symbol**	**HR**	**Log-rank** **p**	**p** **(HR)**

Negative markers	CRP	1.8	3.40E‐04	4.00E‐04
	CDK1	1.4	3.30E‐02	3.40E‐02
	RUNX2	1.4	4.60E‐02	4.70E‐02

Positive markers	ERBB2	0.39	5.50E‐09	1.80E‐08
	IGF1R	0.4	2.30E‐08	6.40E‐08
	TRAF6	0.42	6.20E‐08	1.50E‐07
	CTNND1	0.45	4.90E‐07	9.40E‐07
	ABCG2	0.45	2.80E‐07	5.80E‐07
	EGLN1	0.45	4.50E‐07	8.70E‐07
	RXRA	0.46	1.10E‐06	2.00E‐06
	PPARA	0.47	2.10E‐06	3.50E‐06
	CDH1	0.48	3.70E‐06	5.90E‐06
	SMAD4	0.49	5.40E‐06	8.40E‐06
	SDHC	0.5	1.10E‐05	1.60E‐05
	TLR4	0.53	5.40E‐05	7.10E‐05
	MAPK8	0.54	8.30E‐05	1.10E‐04
	CREB1	0.54	1.20E‐04	1.50E‐04
	SMAD2	0.54	8.40E‐05	1.10E‐04
	NPM1	0.54	8.50E‐05	1.10E‐04
	XIAP	0.54	1.10E‐04	1.40E‐04
	SMARCB1	0.54	1.40E‐04	1.70E‐04
	SMAD3	0.55	1.20E‐04	1.50E‐04
	KITLG	0.55	1.20E‐04	1.50E‐04
	AKT1	0.56	2.30E‐04	2.80E‐04
	PIK3R1	0.56	2.60E‐04	3.10E‐04
	MAP3K5	0.56	2.60E‐04	3.10E‐04
	TGFA	0.56	2.50E‐04	3.00E‐04
	CDC42	0.6	1.00E‐03	1.10E‐03
	BCL2L1	0.61	1.80E‐03	2.00E‐03
	REN	0.61	1.60E‐03	1.70E‐03
	HNF4A	0.62	2.00E‐03	2.20E‐03
	PGK1	0.63	3.70E‐03	4.00E‐03
	MME	0.64	3.90E‐03	4.10E‐03
	SELE	0.65	5.30E‐03	5.60E‐03
	ATG7	0.65	6.60E‐03	7.00E‐03
	NR3C1	0.67	8.50E‐03	8.90E‐03
	CXCR2	0.68	1.40E‐02	1.50E‐02
	HAVCR2	0.68	1.10E‐02	1.20E‐02
	CDKN1A	0.69	1.90E‐02	1.90E‐02
	IGF2	0.7	2.30E‐02	2.40E‐02
	RELA	0.71	3.00E‐02	3.00E‐02

	**B, signature**			
	**Prognostic panel**	**HR**	**Log-rank** **p**	**p** **(HR)**

	CRP + CDK1	1.6	0.0015	0.0017
	CRP + CDK1 + RUNX2	1.7	0.0012	0.0013

Abbreviations: HR, hazard ratio; PIM, protein interaction map; RCCC, renal clear cell carcinoma.

Furthermore, analysis using the Kaplan–Meier plotter indicated that downregulation of has‐miR‐26a‐1‐3p, has‐miR‐144–3P, and has‐miR‐144–5P was significantly associated with poor prognosis in patients with KIRC (Log‐rank test, *p* value < 0.05), as depicted in Supporting Data 9.

Moreover, analysis of the UALCAN database revealed that miR‐26a‐1 expression was significantly downregulated, while has‐miR‐144 was upregulated, in renal carcinoma tissues compared to healthy controls (*p* = 1*E* − 12 and *p* = 2.28*E* − 12, respectively) (Figure 4). These independent findings are consistent with our study’s results.

Figure 4Validation of miRNA expression in KIRC. Analysis from the UALCAN database confirms the significant dysregulation of (a) hsa‐mir‐144 and (b) hsa‐mir‐26a‐1 in primary tumors compared to normal tissue. The box plots display the distribution of miRNA expression (*Y*‐axis, in reads per million) across the different sample groups (*X*‐axis). KIRC, kidney renal clear cell carcinoma.

**Figure:**
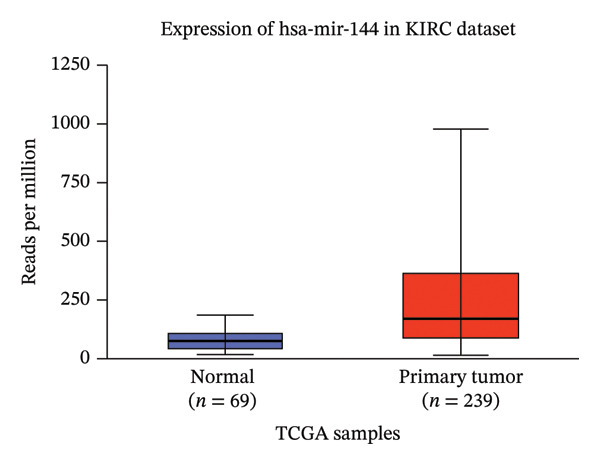
(a)

**Figure:**
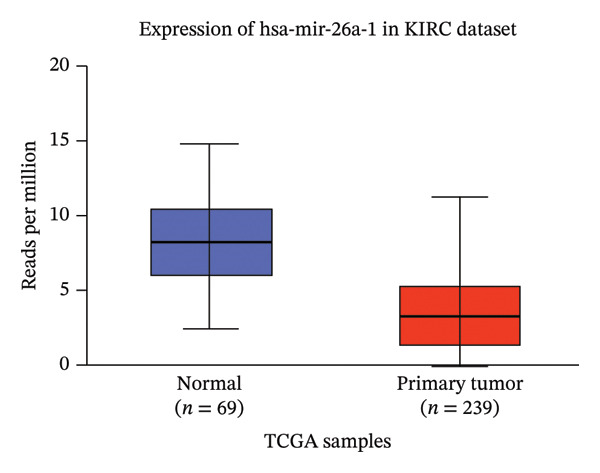
(b)

### 3.4. Gene Expression Pattern and Stage Plot Analyses

Utilizing data from the GEPIA2 database, our gene expression analysis revealed distinct dysregulation patterns of selected biomarkers in RCCC compared to normal kidney tissues. Boxplot analysis indicated that CRP mRNA levels did not substantially elevate RCCC, CDK1, and RUNX2, demonstrating significant upregulation in RCCC tissues.

Moreover, CRP levels showed no statistically significant differences between early and late‐stage RCCC when examining stage‐dependent expression changes. In contrast, CDK1 and RUNX2 showed significant differential expression across tumor stages (Pr (> F) < 0.05), as illustrated in Figure 5. These findings suggest that the transcripts of CDK1 and RUNX2 may serve as valuable indicators of RCCC progression. The observed results underscore CDK1 and RUNX2 as biologically relevant gene expression alterations in the pathogenesis and clinical course of RCCC.

Figure 5The gene expression patterns of prognostic markers, namely, (a) CRP, (b) CDK1, and (c) RUNX2, were analyzed in KIRC in comparison with healthy control kidney tissue. Box plots, derived from a dataset comprising 523 KIRC tissues (depicted in yellow) and 100 healthy kidney tissues (depicted in cyan), were used to represent the expression levels of these markers visually. Stage plot analysis was conducted for (d) CRP, (e) CDK1, and (f) RUNX2. This analysis provides insights into the relationship between marker expression and KIRC stages. KIRC, kidney renal clear cell carcinoma.

**Figure:**
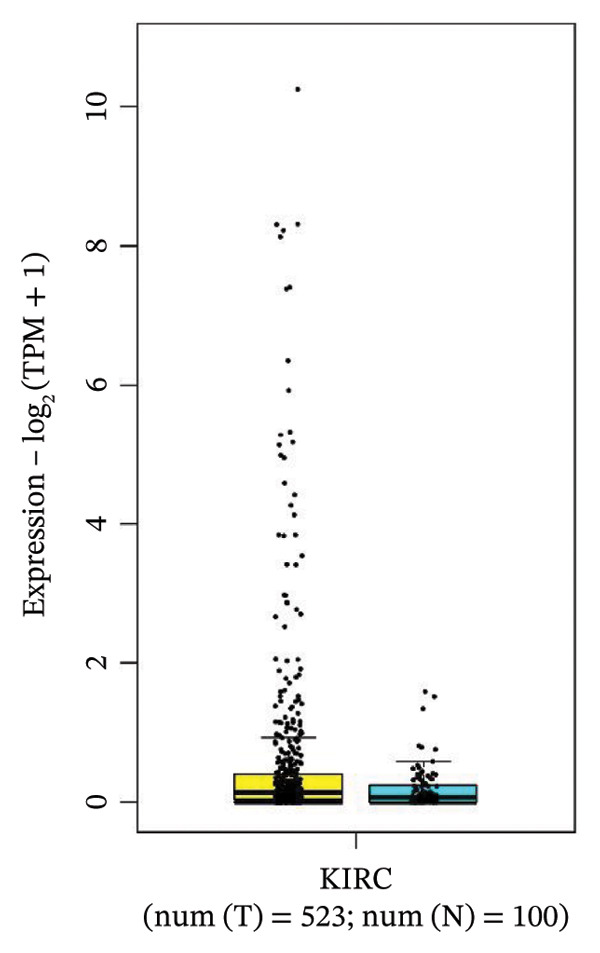
(a)

**Figure:**
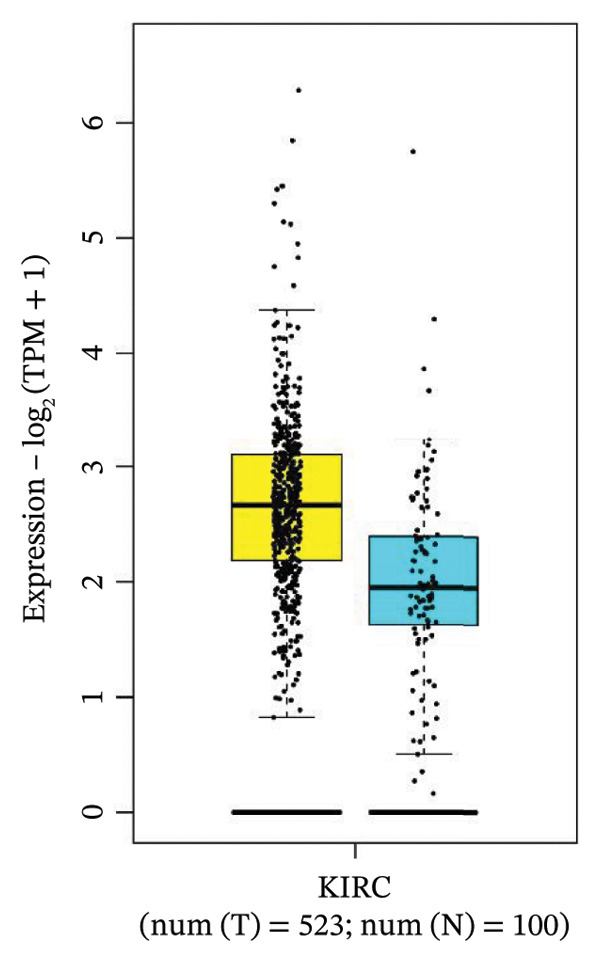
(b)

**Figure:**
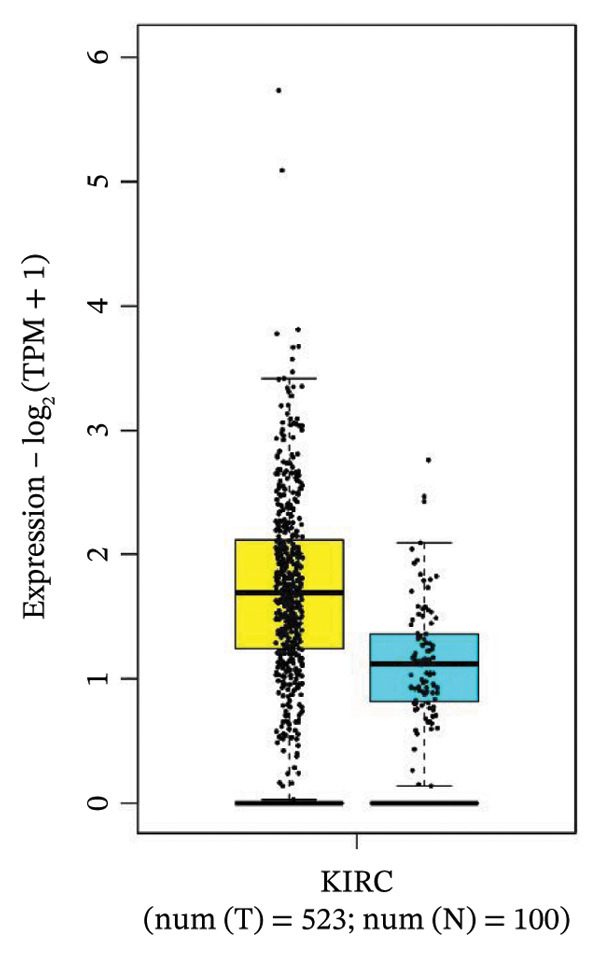
(c)

**Figure:**
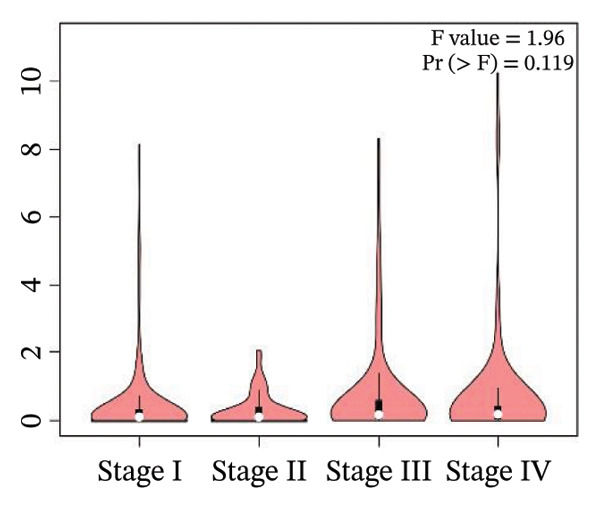
(d)

**Figure:**
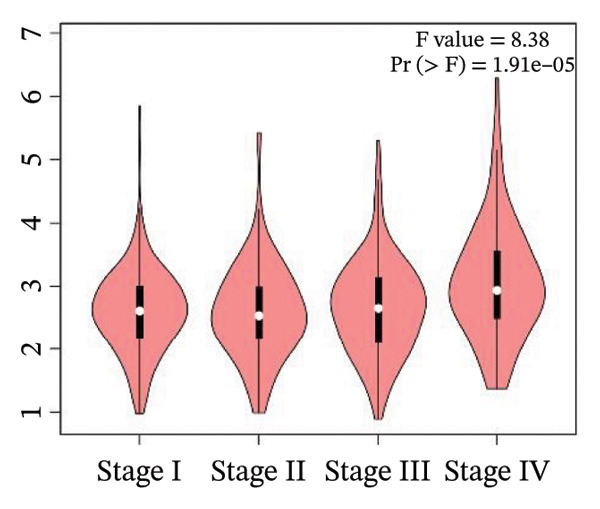
(e)

**Figure:**
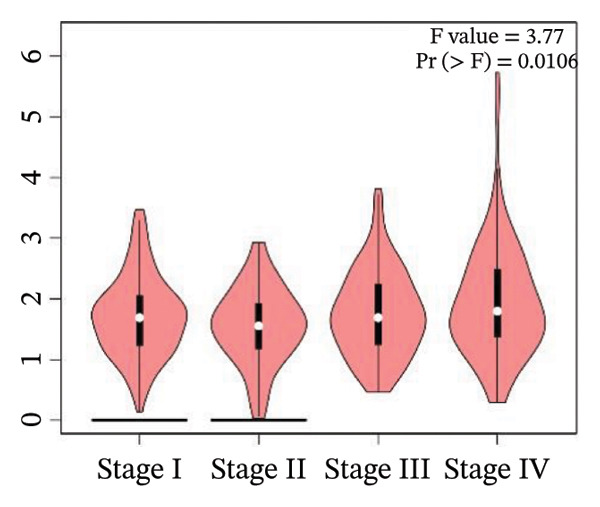
(f)

### 3.5. RT‐PCR

**Figure 6:**
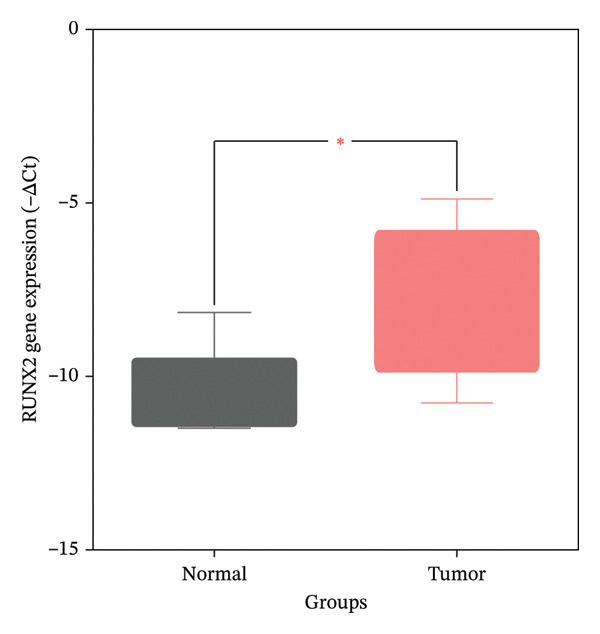
Comparison of RUNX2 gene expression levels between renal cancer and normal renal tissues. ^∗^ Indicated significant difference at *p* value < 0.05.

The expression of the RUNX2 gene in 10 renal tissues (cancerous vs. normal) was investigated using RT‐PCR. As shown in Figure 6, gene expression analysis revealed that RUNX2 expression is significantly higher in renal cancer tissues than in normal renal tissues (*p* value < 0.05).

### 3.6. Independent Validation of RUNX2 and CDK1 Expression and Prognostic Significance

The initial findings of RUNX2 and CDK1 as critical hub genes prompted further validation in independent cohorts. Analysis of the GEO dataset GSE76351 confirmed significant overexpression of both genes in RCCC tissues compared to normal kidney tissues. Specifically, RUNX2 exhibited an FC of 1.89 with an FDR of 2.62E‐06, while CDK1 showed an FC of 1.88 with an FDR of 2.63E‐03.

Subsequent survival analysis using the Kaplan–Meier plotter database, based on RNA‐Seq data, substantiated the poor prognostic role of high RUNX2 and CDK1 expression. Elevated expression of RUNX2 was associated with significantly reduced overall survival (HR = 1.93, Log‐rank *p* = 2.3*E* − 05). Similarly, high CDK1 expression correlated with markedly worse patient outcomes (HR = 2.26, Log‐rank *p* = 8.3*e* − 08) (Supporting Data 10). These results from extensive, independent datasets robustly confirm the protumoral roles of RUNX2 and CDK1 in RCCC pathogenesis and underscore their value as prognostic biomarkers.

### 3.7. Correlation Analysis Reveals Putative miRNA–Target Regulatory Axes

To explore potential associations between prognostic miRNAs and protumoral hub genes, expression correlations were analyzed within the TCGA–KIRC cohort using the starBase platform (Supporting Data 11). Several miRNA–gene pairs exhibited statistically significant inverse correlations; however, the observed correlation coefficients were modest in magnitude.

Specifically, hsa‐miR‐26a‐1‐3p showed a weak but statistically significant negative correlation with RUNX2 expression (*r* = −0.153, *p* = 4.69*e* − 04). Similarly, hsa‐miR‐144‐3p demonstrated weak inverse correlations with RUNX2 (*r* = −0.087, *p* = 4.78*e* − 02) and CDK1 (*r* = −0.089, *p* = 4.32*e* − 02). In contrast, correlations between hsa‐miR‐144–5p and RUNX2 (*r* = −0.054, *p* = 2.18*e* − 01) or CDK1 (*r* = −0.042, *p* = 3.37*e* − 01) were not statistically significant.

Although some of the associations reached statistical significance, their small effect sizes suggest weak correlations, consistent with the multifactorial and context‐dependent nature of miRNA‐mediated gene regulation. These findings indicate potential miRNA–mRNA associations but, alone, do not constitute strong regulatory interactions.

## 4. Discussion

RCCC is one of the most prevalent types of kidney malignancies and a primary histological subtype of renal cancers. A high morbidity rate, poor prognosis, severe clinical manifestations, and metastatic potential characterize this aggressive tumor. As the predominant form of renal neoplasms, RCCC constitutes a significant health burden due to its treatment‐resistant and recurrent nature, which leads to unsatisfactory clinical outcomes [10, 31–33]. Further research into the unique molecular features and microenvironmental cues that sustain RCCC is imperative to identify novel prognostic indicators and actionable therapeutic targets for this deadly disease. Comprehensive insights into the intricate mechanisms that promote RCCC may pave the way for innovative, personalized, precision‐medicine strategies to improve patient survival and quality of life.

Present analyses identified three specific miRNAs—miR‐26a‐1‐3p, miR‐144‐3p, and miR‐144‐5p—that have prognostic value in RCCC. Underexpression of these noncoding RNA transcripts in RCCC tissues correlates with poorer patient outcomes. In addition, further investigation of the RCCC–PIM network identified three hub genes intimately involved in RCCC pathogenesis and progression: C‐reactive protein (CRP), cyclin‐dependent kinase 1 (CDK1), and runt‐related transcription factor 2 (RUNX2). Overexpression of these central genes is also strongly associated with decreased overall survival among RCCC patients. CRP overexpression shows the strongest association with mortality risk (HR = 1.8, *p* value = 0.000340) among the hub genes analyzed. Therefore, dysregulation of these three miRNAs and hub genes may drive RCCC tumor growth and serve as promising prognostic markers or therapeutic candidates, warranting further research.

The regulatory functions of miR‐26a‐1‐3p have been scrutinized across varied cancer subtypes, including renal neoplasms. Du et al. [34] evaluated miR‐26a‐1‐3p expression in metastatic RCC mRCC, revealing significant associations between miR‐26a‐1‐3p levels and overall patient survival. The authors incorporated two independent mRCC patient cohorts, comprising a discovery set (*n* = 44) and a validation set (*n* = 65). Multivariate modeling integrating miR‐26a‐1‐3p expression achieved superior prognostic accuracy over models relying solely on established clinical parameters. These findings position miR‐26a‐1‐3p as a promising tissue biomarker for stratifying mRCC patient outcomes and provide the impetus for larger scale validation of its clinical utility. Elucidating the specific molecular interactions governing miR‐26a‐1‐3p activity in mRCC may yield further insights into disease pathogenesis and targeted therapeutic opportunities.

Emerging evidence, spearheaded by Zhao et al. [35], substantiates a pivotal role for miR‐144‐3p in the molecular pathology of RCC, mediated through crosstalk with the long noncoding RNA NORAD and the proto‐oncogenic MYCN transcript. Mechanistic analyses illuminated NORAD as a molecular sponge sequestering miR‐144‐3p, consequently elevating MYCN expression—an established promoter of migratory and proliferative signaling cascades in neoplastic cells. Expression quantification further indicated that miR‐144‐3p was significantly downregulated in RCC specimens compared with healthy renal tissues, suggesting that miR‐144‐3p may exert tumor‐suppressive effects. Correspondingly, NORAD overexpression enhanced RCC cell lines′ proliferative and invasive capabilities, properties that were mitigated upon miR‐144‐3p co‐upregulation. This coordinated inverse correlation between NORAD and miR‐144‐3p, culminating in miR‐144‐3p release and subsequent MYCN derepression, delineates a cohesive disease‐associated signaling axis that drives RCC pathogenesis. Elucidating the subtler nuances of this crosstalk may further illuminate critical RCC dependencies that warrant therapeutic targeting.

Mounting evidence positions CRP as a putative prognostic marker holding robust correlations with clinical outcomes in RCC. A recent retrospective analysis by Yano et al. [36] evaluated serum CRP levels in patients with RCCC and non–clear cell RCC undergoing dual immunotherapy with ipilimumab and nivolumab. This investigation confirmed the prognostic utility of CRP, showing that levels above 1.0 mg/dL are strongly associated with decreased overall survival in RCCC patients. These findings support the hypothesis that systemic inflammatory activity, as quantified by elevated serum CRP, adversely affects RCC progression. Additional work by Ali et al. [37] linked heightened intratumoral CRP expression to higher Fuhrman grade and pathological stage in RCCC cases, suggesting that CRP upregulation within the tumor microenvironment may directly contribute to disease progression. While a causal relationship remains unconfirmed, this study proffers intratumoral CRP as a candidate tissue biomarker for stratifying RCCC by prognostic risk.

Quantitative phosphoproteomic profiling by Senturk et al. [38] recently provided an extensive characterization of differentially regulated phosphopeptides in RCCC specimens compared with normal kidney tissues. Bioinformatic analyses of the over 600 phosphosites with significant tumor‐associated expression changes revealed enrichment for proteins involved in cellular proliferation and cytoskeletal reorganization—processes closely allied to renal malignancies′ migratory and invasive capacity. Notably, CDK1 emerged as a dominant upstream kinase responsible for phosphorylation of proliferation‐linked substrates. Concordant upregulation of CDK1‐targeted proliferative proteins underscores the pivotal role of aberrant CDK1 signaling in perpetuating the oncogenic phenotype, suggesting CDK1 as a prospective therapeutic target in RCCC. Additional work by Li et al. [39] documented heightened expression of non‐SMC condensin I complex subunit *G* (NCAPG) in RCCC, linking NCAPG overexpression to adverse clinical outcomes, including larger tumor size and poor patient survival. The authors further demonstrated that NCAPG’s stimulation of RCCC growth depends on CDK1‐mediated phosphorylation, thereby substantiating a direct mechanistic association between NCAPG abundance and CDK1 activity. These findings provide actionable insights into CDK1‐driven tumorigenic pathways and support the NCAPG/CDK1 partnership as a potential therapeutic target. Therefore, accumulated evidence for CDK1 underscores its multifaceted involvement across core signaling cascades that enable RCCC pathogenesis, including those governing proliferation and migration. The association between CDK1 dysregulation and an unfavorable survival prognosis in RCCC patients further underscores CDK1’s pivotal role in driving disease progression. This aligns precisely with the established function of CDK1 as a cardinal cell cycle controller, whose unrestrained activity may culminate in the hallmark unchecked cellular proliferation of oncogenesis.

Emerging evidence positions RUNX2 as a critical signaling node orchestrating the complex molecular interactions underlying RCCC pathogenesis. Aberrantly high RUNX2 expression, best known for its physiological roles in osteogenic and chondrogenic differentiation, is recurrent in both human RCCC tumors and derived cell lines. Initial work by Song et al. [40] characterized a tumorigenic partnership between RUNX2 and stearoyl‐CoA desaturase 1 (SCD1), spurring RCCC proliferation and migration through coactivation of oncogenic Wnt/β‐catenin signaling, thus nominating the RUNX2/SCD1 axis as a potential therapeutic target. Follow‐up analyses by Wu et al. [41] linked RUNX2 to an interconnected regulatory loop that also involves the Zic family member 2 (Zic2) and the tumor suppressor nucleolar and coiled‐body phosphoprotein 1 (NOLC1). Here, Zic2‐mediated stimulation of RUNX2 transcription, coupled with RUNX2‐induced NOLC1 suppression, promotes RCCC malignancy and portends a dismal prognosis in human patients. Most recently, Song et al. [42] delineated MAPK11‐catalyzed RUNX2 phosphorylation as the crucial event underlying the stabilized RUNX2 protein abundance in RCCC cells. The resultant accumulation of RUNX2 drives uncontrolled proliferation and invasion, in part by physically associating with active, phosphorylated MAPK11. Also, in the present study, RUNX2 expression in five renal cancer tissues compared to five renal normal tissues was measured using RT‐PCR. Findings showed a significantly higher expression level of RUNX2 in renal cancer tissues than in renal normal tissues.

Further analysis in this study revealed the “PI3K–Akt signaling pathway” (KEGG: 04,151) as highly enriched and significant in the malignant transformation of healthy renal tissue to RCCC, second only to the “Pathways in cancer” (KEGG: 05,200). He et al. [43] focused on the role of Transgelin 2 (TAGLN2) and its impact on RCCC via the PI3K/Akt pathway. They determined that TAGLN2 expression was markedly increased in multiple cancer tissues, especially RCCC, using comprehensive databases and experimental approaches. Elevated TAGLN2 was associated with poor clinical variables and survival prognosis. Silencing TAGLN2 in vitro inhibited processes involved in tumor progression, and these effects were modulated by PI3K/Akt signaling. Similarly, Wang et al. [44] found that Schlafen 11 (SLFN11) overexpression in RCCC was associated with poor outcomes. Knocking down SLFN11 suppressed cell proliferation, migration, and invasion and promoted apoptosis, aligning with PI3K/Akt functions in cancer. These findings reinforce the likely central, functional role of PI3K/Akt in renal carcinogenesis. Targeting this pathway, directly or via associated effectors, could thus provide therapeutic benefit in RCCC.

While this study provides valuable insights into the molecular landscape of RCCC, several limitations should be acknowledged. First, the initial bioinformatics analysis was conducted on a relatively small dataset, which may affect the generalizability of the findings. A larger sample size in the discovery phase could have provided greater statistical power and identified a more comprehensive set of biomarkers.

Among the identified candidates, RUNX2 showed the strongest prognostic relevance and central role in the network; therefore, it was prioritized for RT‐qPCR validation in paired renal tumor and normal tissues. Additional RT‐qPCR validation of CDK1 and hub miRNAs is currently limited mainly by the availability of suitable tissue material and high‐quality RNA/cDNA from the same patients. Expanding the validation would require a separate experimental phase with newly collected samples. Therefore, broader experimental validation is planned as part of a follow‐up study with an expanded cohort.

Finally, while the significant inverse correlation between hsa‐miR‐26a‐1‐3p and RUNX2 provides strong supportive evidence, the regulatory interactions identified in this study through bioinformatics await experimental validation using luciferase reporter assays and Western blot analysis in future work.

## 5. Conclusion

Through comprehensive analyses, 15 DEMs and 75 interactive hub genes were identified as recurrently dysregulated in RCCC tissues. Notably, diminished expression of miR‐26a‐1‐3p, miR‐144‐3p, and miR‐144‐5p was significantly associated with poorer survival outcomes in RCCC patients. The prognostic value of these miRNAs and the protumoral role of the hub genes CDK1 and RUNX2 were further validated in independent GEO and TCGA–KIRC cohorts. Confirmation of RUNX2 overexpression was achieved both in our local patient samples via RT‐PCR and in independent public datasets. Functional enrichment analyses further supported dysregulation of cancer‐associated pathways, including PI3K–Akt and MAPK signaling, apoptosis inhibition, and cell cycle progression. The findings of this study, strengthened by multilevel validation, highlight the intricate interplay between specific prognostic miRNAs, their target hub genes, and enriched signaling cascades, providing deeper insights into RCCC pathogenesis mechanisms. It should be noted that statistically significant miRNA–mRNA correlations in large transcriptomic datasets may reflect subtle regulatory effects rather than strong direct interactions, underscoring the need for functional validation studies. This knowledge bears immense potential for identifying innovative drug targets and developing personalized therapeutic strategies to combat this formidable carcinoma.

## Author Contributions

Conceptualization: A.T. and H.M. Data curation: A.T. and H.M. Formal analysis: A.T., K.M., and H.M. Investigation: A.T. and M.G. Methodology: A.T., M.G., K.M., and H.M. Project administration: A.T. Software: A.T., M.G., K.M., and H.M. Validation: A.T., H.M., and M.G. Visualization: A.T. and H.M. Writing–original draft: A.T., M.G., K.M., and H.M. Writing–review and editing: A.T. and H.M.

## Funding

This research received no specific grant from any funding agency in the public, commercial, or not‐for‐profit sectors.

## Disclosure

All authors read and approved the final version of the manuscript.

## Ethics Statement

The present study was approved by the Ethics Committee of Hamadan University of Medical Sciences, Hamadan, Iran (IR.UMSHA.REC.1403.352).

## Consent

Written informed consent was obtained from all participants for the use of biological samples.

## Conflicts of Interest

The authors declare no conflicts of interest.

## Supporting Information

Supporting Data 1. Primer sequences for real‐time PCR.

Supporting Data 2. A Venn diagram between DEM targets and RCCC‐associated genes retrieved from the DisGeNET database.

Supporting Data 3. Seventy‐four PIM hubs were identified in RCCC patients.

Supporting Data 4. Signaling pathways linked to RCCC.

Supporting Data 5. Biological processes linked to RCCC.

Supporting Data 6. Molecular functions linked to RCCC.

Supporting Data 7. Cellular components linked to RCCC.

Supporting Data 8. Kaplan–Meier survival curves were generated for various biomarkers.

Supporting Data 9. Prognostic role of (a) has‐miR‐26a‐1‐3p, (b) has‐miR‐144–3p, and (c) has‐miR‐144–5p in patients with clear cell renal cell carcinoma.

Supporting Data 10. Overall survival analysis of (a) RUNX2 and (b) CDK1 in KIRC patients was generated using RNA‐Seq data with the Kaplan–Meier plotter tool. Patients were stratified into “high” and “low” expression groups based on the median expression level of each gene. High expression of both RUNX2 (HR = 1.93, Log‐rank *p* = 2.3*e* − 05) and CDK1 (HR = 2.26, Log‐rank *p* = 8.3*e* − 08) is significantly correlated with reduced overall survival, confirming their role as strong negative prognostic biomarkers in KIRC. KIRC, kidney renal clear cell carcinoma.

Supplementary Data 11. Scatter plots depicting the correlation between the expression levels of key miRNAs and hub genes in 517 RCCC samples from the starBase database. The analysis shows the relationship for: (a) hsa‐miR‐144‐3p vs. RUNX2, (b) hsa‐miR‐144‐5p vs. RUNX2, (c) hsa‐miR‐26a‐1‐3p vs. RUNX2, (d) hsa‐miR‐144‐3p vs. CDK1, (e) hsa‐miR‐144‐5p vs. CDK1, and (f) hsa‐miR‐26a‐1‐3p vs. CDK1. KIRC, kidney renal clear cell carcinoma.

## Supporting information


**Supporting Information** Additional supporting information can be found online in the Supporting Information section.

## Data Availability

Data are available upon request due to privacy/ethical restrictions.
