# Supplementary material for: Identifying Prognostic Biomarkers and Key Pathways in Renal Clear Cell Carcinoma: A Pilot Study Using Integrated miRNA and Gene Expression Analysis
Source: Biochem Res Int. 2026 Feb 26;2026:3213941. doi: 10.1155/bri/3213941 (PMC12945469; doi:10.1155/bri/3213941)
Supplement: Supplementary file 1 — Supporting Information Additional supporting information can be found online in the Supporting Information section. [file BRI-2026-3213941-s001.docx]

**Supplementary Data 1.** Primer sequences for Real-Time PCR.

| **Gene Symbols** | **Forward (5՜ to 3՜)** | **Reverse (5՜ to 3՜)** |
| --- | --- | --- |
| **GAPDH** | AAGGCTGTGGGCAAGGTCATC | GCGTCAAAGGTGGAGGAGTGG |
| **RUNX2** | TGTTCTGTGGTTTCATAGTTAAGC | CCCTGTTGTGTTGTTTGGTAAG |

PCR, polymerase chain reaction.

**
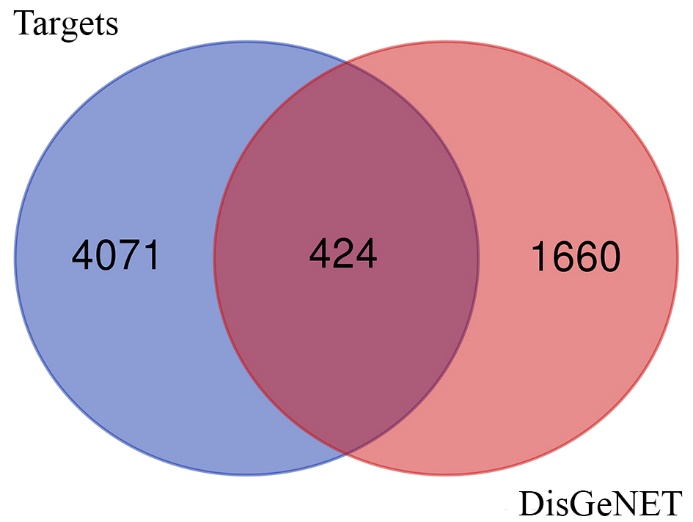
**

**Supplementary Data 2.** A Venn diagram between DEM-targets and RCCC-associated genes retrieved from the DisGeNET database. RCCC, Renal clear cell carcinoma.

**Supplementary Data 3.** Seventy-four PIM hubs were identified in RCCC patients.

| **Name** | **Degree** | **Betweenness** |
| --- | --- | --- |
| TP53 | 171 | 0.142 |
| AKT1 | 170 | 0.112 |
| MYC | 148 | 0.089 |
| EGFR | 143 | 0.073 |
| SRC | 126 | 0.072 |
| VEGFA | 126 | 0.047 |
| EGF | 107 | 0.025 |
| IL1B | 106 | 0.040 |
| CDH1 | 99 | 0.030 |
| ERBB2 | 97 | 0.016 |
| FGF2 | 75 | 0.009 |
| TLR4 | 73 | 0.021 |
| BCL2L1 | 70 | 0.008 |
| MDM2 | 66 | 0.014 |
| ICAM1 | 66 | 0.007 |
| CDC42 | 64 | 0.024 |
| MAPK8 | 64 | 0.007 |
| IGF1R | 64 | 0.005 |
| PIK3R1 | 63 | 0.010 |
| SMAD4 | 62 | 0.014 |
| BRCA1 | 57 | 0.008 |
| CXCL12 | 57 | 0.007 |
| MMP2 | 57 | 0.005 |
| CDKN1A | 57 | 0.004 |
| CREB1 | 56 | 0.013 |
| SMAD3 | 56 | 0.008 |
| RELA | 56 | 0.007 |
| CASP8 | 56 | 0.006 |
| TRAF6 | 50 | 0.019 |
| NANOG | 50 | 0.019 |
| RPS6KB1 | 50 | 0.010 |
| RUNX2 | 48 | 0.020 |
| SMAD2 | 48 | 0.006 |
| CD40 | 47 | 0.008 |
| CD28 | 44 | 0.011 |
| PXN | 44 | 0.004 |
| PRKCA | 43 | 0.012 |
| KITLG | 43 | 0.008 |
| CDK1 | 43 | 0.007 |
| NPM1 | 42 | 0.010 |
| NR3C1 | 42 | 0.009 |
| IGF2 | 42 | 0.008 |
| CSF1R | 41 | 0.005 |
| HNF4A | 40 | 0.015 |
| NCAM1 | 40 | 0.014 |
| PGR | 40 | 0.005 |
| CRP | 38 | 0.006 |
| PPARA | 37 | 0.005 |
| XIAP | 35 | 0.006 |
| ANXA2 | 33 | 0.009 |
| REN | 33 | 0.006 |
| SDHC | 32 | 0.008 |
| CTNND1 | 32 | 0.005 |
| SELE | 31 | 0.009 |
| CTSB | 30 | 0.011 |
| TFRC | 30 | 0.011 |
| MAP3K5 | 30 | 0.008 |
| TGFA | 29 | 0.007 |
| SOD2 | 29 | 0.007 |
| RASSF1 | 28 | 0.010 |
| MME | 28 | 0.004 |
| ABCG2 | 27 | 0.009 |
| ATG7 | 25 | 0.011 |
| HK2 | 25 | 0.011 |
| SFRP1 | 25 | 0.009 |
| SMARCB1 | 25 | 0.007 |
| CXCR2 | 23 | 0.006 |
| HAVCR2 | 23 | 0.005 |
| YBX1 | 23 | 0.005 |
| EGLN1 | 22 | 0.007 |
| AGO2 | 22 | 0.004 |
| RXRA | 21 | 0.007 |
| VHL | 20 | 0.010 |
| PGK1 | 19 | 0.005 |

PIM, protein interaction map; RCCC, Renal clear cell carcinoma.

**Supplementary Data 4.** Signaling pathways linked to RCCC.

| **Cluster no.** | **Pathway ID** | **Pathway name** | **FDR** |
| --- | --- | --- | --- |
| 1 | KEGG:05200 | Pathways in cancer | 5.82E-16 |
|  | KEGG:04151 | PI3K-Akt signaling pathway | 3.48E-15 |
|  | KEGG:04010 | MAPK signaling pathway | 2.60E-14 |
|  | KEGG:05205 | Proteoglycans in cancer | 5.34E-12 |
|  | KEGG:04015 | Rap1 signaling pathway | 2.21E-09 |
|  | REAC:R-HSA-9006934 | Signaling by Receptor Tyrosine Kinases | 4.04E-09 |
|  | KEGG:05210 | Colorectal cancer | 8.91E-09 |
|  | KEGG:04014 | Ras signaling pathway | 9.46E-09 |
|  | KEGG:04668 | TNF signaling pathway | 1.38E-07 |
|  | REAC:R-HSA-1280215 | Cytokine Signaling in Immune system | 1.63E-07 |
|  | KEGG:05161 | Hepatitis B | 3.07E-07 |
|  | KEGG:05417 | Lipid and atherosclerosis | 5.20E-07 |
|  | KEGG:05166 | Human T-cell leukemia virus 1 infection | 6.76E-07 |
|  | REAC:R-HSA-449147 | Signaling by Interleukins | 1.4581E-06 |
|  | KEGG:05224 | Breast cancer | 1.7903E-06 |
|  | KEGG:05226 | Gastric cancer | 1.9109E-06 |
|  | KEGG:05225 | Hepatocellular carcinoma | 5.7219E-06 |
|  | REAC:R-HSA-1257604 | PIP3 activates AKT signaling | 9.9467E-06 |
|  | REAC:R-HSA-162582 | Signal Transduction | 1.6751E-05 |
|  | KEGG:04510 | Focal adhesion | 3.6091E-05 |
|  | REAC:R-HSA-212436 | Generic Transcription Pathway | 4.1564E-05 |
|  | REAC:R-HSA-9006925 | Intracellular signaling by second messengers | 4.4871E-05 |
|  | KEGG:05207 | Chemical carcinogenesis - receptor activation | 5.3997E-05 |
|  | KEGG:05163 | Human cytomegalovirus infection | 8.9722E-05 |
|  | REAC:R-HSA-73857 | RNA Polymerase II Transcription | 0.00020485 |
|  | REAC:R-HSA-74160 | Gene expression (Transcription) | 0.00100079 |
|  | REAC:R-HSA-5663202 | Diseases of signal transduction by growth factor receptors and second messengers | 0.00152042 |
|  | REAC:R-HSA-168256 | Immune System | 0.00364214 |
|  | REAC:R-HSA-1643685 | Disease | 0.04943189 |
| 2 | KEGG:05200 | Pathways in cancer | 5.82E-16 |
|  | KEGG:04151 | PI3K-Akt signaling pathway | 3.48E-15 |
|  | KEGG:04010 | MAPK signaling pathway | 2.60E-14 |
|  | KEGG:05212 | Pancreatic cancer | 9.90E-14 |
|  | KEGG:05205 | Proteoglycans in cancer | 5.34E-12 |
|  | KEGG:04015 | Rap1 signaling pathway | 2.21E-09 |
|  | REAC:R-HSA-9006934 | Signaling by Receptor Tyrosine Kinases | 4.04E-09 |
|  | KEGG:05210 | Colorectal cancer | 8.91E-09 |
|  | KEGG:04014 | Ras signaling pathway | 9.46E-09 |
|  | KEGG:04668 | TNF signaling pathway | 1.38E-07 |
|  | REAC:R-HSA-1280215 | Cytokine Signaling in Immune system | 1.63E-07 |
|  | KEGG:05161 | Hepatitis B | 3.07E-07 |
|  | KEGG:05417 | Lipid and atherosclerosis | 5.20E-07 |
|  | KEGG:05166 | Human T-cell leukemia virus 1 infection | 6.76E-07 |
|  | REAC:R-HSA-449147 | Signaling by Interleukins | 1.4581E-06 |
|  | KEGG:05224 | Breast cancer | 1.7903E-06 |
|  | KEGG:05226 | Gastric cancer | 1.9109E-06 |
|  | KEGG:05225 | Hepatocellular carcinoma | 5.7219E-06 |
|  | REAC:R-HSA-1257604 | PIP3 activates AKT signaling | 9.9467E-06 |
|  | REAC:R-HSA-162582 | Signal Transduction | 1.6751E-05 |
|  | KEGG:04510 | Focal adhesion | 3.6091E-05 |
|  | REAC:R-HSA-212436 | Generic Transcription Pathway | 4.1564E-05 |
|  | REAC:R-HSA-9006925 | Intracellular signaling by second messengers | 4.4871E-05 |
|  | KEGG:05207 | Chemical carcinogenesis - receptor activation | 5.3997E-05 |
|  | KEGG:05163 | Human cytomegalovirus infection | 8.9722E-05 |
|  | REAC:R-HSA-73857 | RNA Polymerase II Transcription | 0.00020485 |
|  | REAC:R-HSA-74160 | Gene expression (Transcription) | 0.00100079 |
|  | REAC:R-HSA-5663202 | Diseases of signal transduction by growth factor receptors and second messengers | 0.00152042 |
|  | REAC:R-HSA-168256 | Immune System | 0.00364214 |
|  | REAC:R-HSA-1643685 | Disease | 0.04943189 |
| 4 | REAC:R-HSA-162582 | Signal Transduction | 1.12E-08 |
|  | REAC:R-HSA-5663202 | Diseases of signal transduction by growth factor receptors and second messengers | 8.83E-07 |
|  | REAC:R-HSA-1280215 | Cytokine Signaling in Immune system | 0.00015621 |
|  | REAC:R-HSA-1643685 | Disease | 0.00025326 |

RCCC, Renal clear cell carcinoma..

**Supplementary Data 5.** Biological processes linked to RCCC.

| **Closter no.** | **Term ID** | **Term name** | **FDR** |
| --- | --- | --- | --- |
| 1 | GO:0043067 | regulation of programmed cell death | 3.63E-22 |
|  | GO:0009966 | regulation of signal transduction | 7.84E-22 |
|  | GO:0042981 | regulation of apoptotic process | 2.91E-21 |
|  | GO:0023051 | regulation of signaling | 9.21E-21 |
|  | GO:0048583 | regulation of response to stimulus | 9.64E-21 |
|  | GO:0010646 | regulation of cell communication | 1.03E-20 |
|  | GO:0009719 | response to endogenous stimulus | 1.73E-20 |
|  | GO:0035556 | intracellular signal transduction | 3.06E-20 |
|  | GO:0048522 | positive regulation of cellular process | 7.20E-20 |
|  | GO:1902533 | positive regulation of intracellular signal transduction | 7.30E-20 |
|  | GO:0051247 | positive regulation of protein metabolic process | 1.62E-19 |
|  | GO:0012501 | programmed cell death | 1.64E-19 |
|  | GO:0008219 | cell death | 1.81E-19 |
|  | GO:0042127 | regulation of cell population proliferation | 2.58E-19 |
|  | GO:0007166 | cell surface receptor signaling pathway | 3.34E-19 |
|  | GO:0006915 | apoptotic process | 8.12E-19 |
|  | GO:0009967 | positive regulation of signal transduction | 2.66E-18 |
|  | GO:1902531 | regulation of intracellular signal transduction | 4.75E-18 |
|  | GO:0048518 | positive regulation of biological process | 5.73E-18 |
|  | GO:0048584 | positive regulation of response to stimulus | 6.23E-18 |
|  | GO:0030335 | positive regulation of cell migration | 6.92E-18 |
|  | GO:0023056 | positive regulation of signaling | 8.69E-18 |
|  | GO:0010647 | positive regulation of cell communication | 8.69E-18 |
|  | GO:0051173 | positive regulation of nitrogen compound metabolic process | 1.24E-17 |
|  | GO:2000147 | positive regulation of cell motility | 1.93E-17 |
|  | GO:0070887 | cellular response to chemical stimulus | 2.25E-17 |
|  | GO:0040017 | positive regulation of locomotion | 3.41E-17 |
|  | GO:0051716 | cellular response to stimulus | 3.87E-17 |
|  | GO:0008283 | cell population proliferation | 6.86E-17 |
|  | GO:0051246 | regulation of protein metabolic process | 7.83E-17 |
|  | GO:0042221 | response to chemical | 8.14E-17 |
|  | GO:0010604 | positive regulation of macromolecule metabolic process | 9.46E-17 |
|  | GO:0010562 | positive regulation of phosphorus metabolic process | 1.09E-16 |
|  | GO:0045937 | positive regulation of phosphate metabolic process | 1.09E-16 |
|  | GO:0097190 | apoptotic signaling pathway | 1.35E-16 |
|  | GO:0070848 | response to growth factor | 1.46E-16 |
|  | GO:0007165 | signal transduction | 1.68E-16 |
|  | GO:0050793 | regulation of developmental process | 2.18E-16 |
|  | GO:0071495 | cellular response to endogenous stimulus | 2.54E-16 |
|  | GO:0050790 | regulation of catalytic activity | 3.77E-16 |
|  | GO:0007167 | enzyme-linked receptor protein signaling pathway | 4.19E-16 |
|  | GO:0065009 | regulation of molecular function | 4.85E-16 |
|  | GO:0043085 | positive regulation of catalytic activity | 5.43E-16 |
|  | GO:0050896 | response to stimulus | 6.37E-16 |
|  | GO:0001934 | positive regulation of protein phosphorylation | 7.95E-16 |
|  | GO:0008284 | positive regulation of cell population proliferation | 9.87E-16 |
|  | GO:0071363 | cellular response to growth factor stimulus | 1.20E-15 |
|  | GO:0009893 | positive regulation of metabolic process | 2.34E-15 |
|  | GO:0051347 | positive regulation of transferase activity | 2.75E-15 |
|  | GO:0009653 | anatomical structure morphogenesis | 3.44E-15 |
|  | GO:0042327 | positive regulation of phosphorylation | 5.27E-15 |
|  | GO:0023052 | signaling | 5.36E-15 |
|  | GO:0035295 | tube development | 7.57E-15 |
|  | GO:0044093 | positive regulation of molecular function | 7.91E-15 |
|  | GO:0007154 | cell communication | 1.07E-14 |
|  | GO:0009628 | response to abiotic stimulus | 1.22E-14 |
|  | GO:0035239 | tube morphogenesis | 1.25E-14 |
|  | GO:0043410 | positive regulation of MAPK cascade | 1.32E-14 |
|  | GO:0000165 | MAPK cascade | 1.55E-14 |
|  | GO:0043408 | regulation of MAPK cascade | 1.73E-14 |
|  | GO:0031401 | positive regulation of protein modification process | 2.23E-14 |
|  | GO:0033674 | positive regulation of kinase activity | 4.02E-14 |
|  | GO:0030334 | regulation of cell migration | 5.57E-14 |
|  | GO:0045860 | positive regulation of protein kinase activity | 5.85E-14 |
|  | GO:0007169 | transmembrane receptor protein tyrosine kinase signaling pathway | 6.43E-14 |
|  | GO:0031325 | positive regulation of cellular metabolic process | 7.30E-14 |
|  | GO:0036293 | response to decreased oxygen levels | 7.84E-14 |
|  | GO:0051338 | regulation of transferase activity | 9.27E-14 |
|  | GO:0048731 | system development | 1.12E-13 |
|  | GO:0010033 | response to organic substance | 1.14E-13 |
|  | GO:2000145 | regulation of cell motility | 2.18E-13 |
|  | GO:0019220 | regulation of phosphate metabolic process | 2.67E-13 |
|  | GO:0051174 | regulation of phosphorus metabolic process | 2.72E-13 |
|  | GO:0043066 | negative regulation of apoptotic process | 2.92E-13 |
|  | GO:0070482 | response to oxygen levels | 3.38E-13 |
|  | GO:0001932 | regulation of protein phosphorylation | 3.84E-13 |
|  | GO:0051171 | regulation of nitrogen compound metabolic process | 4.19E-13 |
|  | GO:0040012 | regulation of locomotion | 5.28E-13 |
|  | GO:0043069 | negative regulation of programmed cell death | 5.48E-13 |
|  | GO:1901652 | response to peptide | 5.54E-13 |
|  | GO:0051094 | positive regulation of developmental process | 6.51E-13 |
|  | GO:0034097 | response to cytokine | 6.58E-13 |
|  | GO:1901564 | organonitrogen compound metabolic process | 7.25E-13 |
|  | GO:0051239 | regulation of multicellular organismal process | 9.17E-13 |
|  | GO:0019538 | protein metabolic process | 9.39E-13 |
|  | GO:0001666 | response to hypoxia | 1.01E-12 |
|  | GO:0006468 | protein phosphorylation | 1.16E-12 |
|  | GO:0071310 | cellular response to organic substance | 1.32E-12 |
|  | GO:0097191 | extrinsic apoptotic signaling pathway | 1.34E-12 |
|  | GO:0080090 | regulation of primary metabolic process | 1.49E-12 |
|  | GO:0050673 | epithelial cell proliferation | 1.51E-12 |
|  | GO:0050678 | regulation of epithelial cell proliferation | 1.73E-12 |
|  | GO:0048732 | gland development | 1.75E-12 |
|  | GO:0031399 | regulation of protein modification process | 2.38E-12 |
|  | GO:0048523 | negative regulation of cellular process | 2.53E-12 |
|  | GO:0048513 | animal organ development | 2.54E-12 |
|  | GO:0009725 | response to hormone | 2.63E-12 |
|  | GO:0007275 | multicellular organism development | 2.82E-12 |
|  | GO:0042325 | regulation of phosphorylation | 3.11E-12 |
|  | GO:2001233 | regulation of apoptotic signaling pathway | 4.17E-12 |
|  | GO:0002376 | immune system process | 7.08E-12 |
|  | GO:0006950 | response to stress | 7.32E-12 |
|  | GO:0043549 | regulation of kinase activity | 1.36E-11 |
|  | GO:0032501 | multicellular organismal process | 1.60E-11 |
|  | GO:0045859 | regulation of protein kinase activity | 1.73E-11 |
|  | GO:0030154 | cell differentiation | 2.19E-11 |
|  | GO:0048869 | cellular developmental process | 2.21E-11 |
|  | GO:0040011 | locomotion | 2.24E-11 |
|  | GO:0048856 | anatomical structure development | 2.79E-11 |
|  | GO:2000026 | regulation of multicellular organismal development | 3.31E-11 |
|  | GO:0048519 | negative regulation of biological process | 3.90E-11 |
|  | GO:0072359 | circulatory system development | 4.10E-11 |
|  | GO:0016310 | phosphorylation | 4.22E-11 |
|  | GO:0022612 | gland morphogenesis | 4.81E-11 |
|  | GO:0060255 | regulation of macromolecule metabolic process | 9.97E-11 |
|  | GO:0032502 | developmental process | 1.04E-10 |
|  | GO:0016477 | cell migration | 1.11E-10 |
|  | GO:0010557 | positive regulation of macromolecule biosynthetic process | 1.47E-10 |
|  | GO:0071902 | positive regulation of protein serine/threonine kinase activity | 1.53E-10 |
|  | GO:0019222 | regulation of metabolic process | 2.26E-10 |
|  | GO:0071345 | cellular response to cytokine stimulus | 2.54E-10 |
|  | GO:0031328 | positive regulation of cellular biosynthetic process | 3.47E-10 |
|  | GO:1901698 | response to nitrogen compound | 3.78E-10 |
|  | GO:0040008 | regulation of growth | 4.01E-10 |
|  | GO:0009891 | positive regulation of biosynthetic process | 4.18E-10 |
|  | GO:2001236 | regulation of extrinsic apoptotic signaling pathway | 5.58E-10 |
|  | GO:0080134 | regulation of response to stress | 5.91E-10 |
|  | GO:0031323 | regulation of cellular metabolic process | 6.70E-10 |
|  | GO:1901700 | response to oxygen-containing compound | 6.85E-10 |
|  | GO:0043406 | positive regulation of MAP kinase activity | 8.38E-10 |
|  | GO:0051240 | positive regulation of multicellular organismal process | 9.72E-10 |
|  | GO:0018108 | peptidyl-tyrosine phosphorylation | 1.04E-09 |
|  | GO:0018212 | peptidyl-tyrosine modification | 1.13E-09 |
|  | GO:0009888 | tissue development | 1.24E-09 |
|  | GO:0010628 | positive regulation of gene expression | 1.41E-09 |
|  | GO:0009887 | animal organ morphogenesis | 1.64E-09 |
|  | GO:0045785 | positive regulation of cell adhesion | 1.76E-09 |
|  | GO:0048870 | cell motility | 1.80E-09 |
|  | GO:0040007 | growth | 2.12E-09 |
|  | GO:0045893 | positive regulation of DNA-templated transcription | 2.41E-09 |
|  | GO:0018193 | peptidyl-amino acid modification | 2.61E-09 |
|  | GO:1902680 | positive regulation of RNA biosynthetic process | 2.64E-09 |
|  | GO:0051336 | regulation of hydrolase activity | 2.76E-09 |
|  | GO:0009605 | response to external stimulus | 2.84E-09 |
|  | GO:0006796 | phosphate-containing compound metabolic process | 3.06E-09 |
|  | GO:0060429 | epithelium development | 3.13E-09 |
|  | GO:0001568 | blood vessel development | 3.38E-09 |
|  | GO:0006793 | phosphorus metabolic process | 3.87E-09 |
|  | GO:0030879 | mammary gland development | 4.59E-09 |
|  | GO:0009968 | negative regulation of signal transduction | 5.35E-09 |
|  | GO:1901701 | cellular response to oxygen-containing compound | 5.43E-09 |
|  | GO:0048468 | cell development | 5.77E-09 |
|  | GO:0001944 | vasculature development | 6.41E-09 |
|  | GO:0033002 | muscle cell proliferation | 9.88E-09 |
|  | GO:0048585 | negative regulation of response to stimulus | 1.00E-08 |
|  | GO:0050679 | positive regulation of epithelial cell proliferation | 1.10E-08 |
|  | GO:0002682 | regulation of immune system process | 1.17E-08 |
|  | GO:0048646 | anatomical structure formation involved in morphogenesis | 1.23E-08 |
|  | GO:0010243 | response to organonitrogen compound | 1.29E-08 |
|  | GO:0030155 | regulation of cell adhesion | 1.37E-08 |
|  | GO:0051254 | positive regulation of RNA metabolic process | 1.41E-08 |
|  | GO:0045935 | positive regulation of nucleobase-containing compound metabolic process | 1.53E-08 |
|  | GO:1901699 | cellular response to nitrogen compound | 2.23E-08 |
|  | GO:0023057 | negative regulation of signaling | 2.60E-08 |
|  | GO:0010648 | negative regulation of cell communication | 2.60E-08 |
|  | GO:0050794 | regulation of cellular process | 2.73E-08 |
|  | GO:0045595 | regulation of cell differentiation | 3.30E-08 |
|  | GO:0052548 | regulation of endopeptidase activity | 4.32E-08 |
|  | GO:0050900 | leukocyte migration | 4.37E-08 |
|  | GO:0071396 | cellular response to lipid | 5.44E-08 |
|  | GO:0043065 | positive regulation of apoptotic process | 8.06E-08 |
|  | GO:0022603 | regulation of anatomical structure morphogenesis | 8.33E-08 |
|  | GO:0043405 | regulation of MAP kinase activity | 8.42E-08 |
|  | GO:0051128 | regulation of cellular component organization | 9.25E-08 |
|  | GO:0043434 | response to peptide hormone | 9.97E-08 |
|  | GO:0043281 | regulation of cysteine-type endopeptidase activity involved in apoptotic process | 1.10E-07 |
|  | GO:0002009 | morphogenesis of an epithelium | 1.12E-07 |
|  | GO:0071417 | cellular response to organonitrogen compound | 1.20E-07 |
|  | GO:0001525 | angiogenesis | 1.27E-07 |
|  | GO:0043068 | positive regulation of programmed cell death | 1.34E-07 |
|  | GO:0043412 | macromolecule modification | 1.42E-07 |
|  | GO:0001817 | regulation of cytokine production | 1.51E-07 |
|  | GO:0001816 | cytokine production | 1.70E-07 |
|  | GO:0033993 | response to lipid | 1.71E-07 |
|  | GO:0014070 | response to organic cyclic compound | 1.83E-07 |
|  | GO:0071900 | regulation of protein serine/threonine kinase activity | 1.96E-07 |
|  | GO:1901653 | cellular response to peptide | 2.46E-07 |
|  | GO:0033554 | cellular response to stress | 2.64E-07 |
|  | GO:0050789 | regulation of biological process | 3.02E-07 |
|  | GO:0045944 | positive regulation of transcription by RNA polymerase II | 3.31E-07 |
|  | GO:2000116 | regulation of cysteine-type endopeptidase activity | 5.20E-07 |
|  | GO:0052547 | regulation of peptidase activity | 8.05E-07 |
|  | GO:0007159 | leukocyte cell-cell adhesion | 9.29E-07 |
|  | GO:0048514 | blood vessel morphogenesis | 9.97E-07 |
|  | GO:0097193 | intrinsic apoptotic signaling pathway | 1.011E-06 |
|  | GO:0007155 | cell adhesion | 1.0683E-06 |
|  | GO:0036211 | protein modification process | 1.0823E-06 |
|  | GO:0002684 | positive regulation of immune system process | 1.2725E-06 |
|  | GO:0070371 | ERK1 and ERK2 cascade | 1.2761E-06 |
|  | GO:0048729 | tissue morphogenesis | 1.333E-06 |
|  | GO:0071214 | cellular response to abiotic stimulus | 1.3625E-06 |
|  | GO:0104004 | cellular response to environmental stimulus | 1.3625E-06 |
|  | GO:0050730 | regulation of peptidyl-tyrosine phosphorylation | 1.4343E-06 |
|  | GO:0065007 | biological regulation | 1.6279E-06 |
|  | GO:0032101 | regulation of response to external stimulus | 1.9417E-06 |
|  | GO:0048589 | developmental growth | 2.0742E-06 |
|  | GO:0060562 | epithelial tube morphogenesis | 2.13E-06 |
|  | GO:0045927 | positive regulation of growth | 2.2036E-06 |
|  | GO:0006952 | defense response | 2.6688E-06 |
|  | GO:0070661 | leukocyte proliferation | 2.9843E-06 |
|  | GO:0051345 | positive regulation of hydrolase activity | 3.2353E-06 |
|  | GO:0070663 | regulation of leukocyte proliferation | 3.32E-06 |
|  | GO:0030097 | hemopoiesis | 3.3615E-06 |
|  | GO:0006954 | inflammatory response | 4.2076E-06 |
|  | GO:0006935 | chemotaxis | 4.3827E-06 |
|  | GO:0042330 | taxis | 4.6016E-06 |
|  | GO:1903037 | regulation of leukocyte cell-cell adhesion | 4.7771E-06 |
|  | GO:0032870 | cellular response to hormone stimulus | 8.2288E-06 |
|  | GO:0044419 | biological process involved in interspecies interaction between organisms | 8.8223E-06 |
|  | GO:0042592 | homeostatic process | 9.3744E-06 |
|  | GO:0031324 | negative regulation of cellular metabolic process | 1.0025E-05 |
|  | GO:0070372 | regulation of ERK1 and ERK2 cascade | 1.0258E-05 |
|  | GO:0002521 | leukocyte differentiation | 1.1258E-05 |
|  | GO:0010631 | epithelial cell migration | 1.2412E-05 |
|  | GO:0090132 | epithelium migration | 1.3632E-05 |
|  | GO:0060326 | cell chemotaxis | 1.4062E-05 |
|  | GO:0090130 | tissue migration | 1.6396E-05 |
|  | GO:0032943 | mononuclear cell proliferation | 1.6396E-05 |
|  | GO:0046649 | lymphocyte activation | 1.8145E-05 |
|  | GO:0001822 | kidney development | 1.8505E-05 |
|  | GO:0042060 | wound healing | 2.0594E-05 |
|  | GO:0080135 | regulation of cellular response to stress | 2.331E-05 |
|  | GO:0045321 | leukocyte activation | 2.4532E-05 |
|  | GO:0072001 | renal system development | 2.4863E-05 |
|  | GO:0048545 | response to steroid hormone | 2.4863E-05 |
|  | GO:0009611 | response to wounding | 3.0206E-05 |
|  | GO:0051130 | positive regulation of cellular component organization | 3.0801E-05 |
|  | GO:0042110 | T cell activation | 3.0822E-05 |
|  | GO:0042063 | gliogenesis | 3.1271E-05 |
|  | GO:0071407 | cellular response to organic cyclic compound | 3.7628E-05 |
|  | GO:0019216 | regulation of lipid metabolic process | 4.0193E-05 |
|  | GO:0030162 | regulation of proteolysis | 4.304E-05 |
|  | GO:0033043 | regulation of organelle organization | 5.4431E-05 |
|  | GO:0045597 | positive regulation of cell differentiation | 5.7954E-05 |
|  | GO:0022407 | regulation of cell-cell adhesion | 7.1415E-05 |
|  | GO:0009892 | negative regulation of metabolic process | 8.4857E-05 |
|  | GO:0006955 | immune response | 9.3268E-05 |
|  | GO:0009607 | response to biotic stimulus | 9.8849E-05 |
|  | GO:0001819 | positive regulation of cytokine production | 0.0001023 |
|  | GO:0001775 | cell activation | 0.0001601 |
|  | GO:0065008 | regulation of biological quality | 0.0001647 |
|  | GO:0006355 | regulation of DNA-templated transcription | 0.00018871 |
|  | GO:0098609 | cell-cell adhesion | 0.00019671 |
|  | GO:2001141 | regulation of RNA biosynthetic process | 0.00021665 |
|  | GO:0060537 | muscle tissue development | 0.00022828 |
|  | GO:0051052 | regulation of DNA metabolic process | 0.00023058 |
|  | GO:0009790 | embryo development | 0.00025117 |
|  | GO:0010468 | regulation of gene expression | 0.0002716 |
|  | GO:0019219 | regulation of nucleobase-containing compound metabolic process | 0.00028095 |
|  | GO:0001558 | regulation of cell growth | 0.0002912 |
|  | GO:0009314 | response to radiation | 0.00029761 |
|  | GO:0009410 | response to xenobiotic stimulus | 0.00030415 |
|  | GO:0001667 | ameboidal-type cell migration | 0.0003176 |
|  | GO:0009890 | negative regulation of biosynthetic process | 0.00038797 |
|  | GO:0006351 | DNA-templated transcription | 0.00039628 |
|  | GO:0051707 | response to other organism | 0.00041959 |
|  | GO:0043207 | response to external biotic stimulus | 0.00043155 |
|  | GO:0010605 | negative regulation of macromolecule metabolic process | 0.00048559 |
|  | GO:0032774 | RNA biosynthetic process | 0.00048951 |
|  | GO:0010556 | regulation of macromolecule biosynthetic process | 0.00049795 |
|  | GO:0006357 | regulation of transcription by RNA polymerase II | 0.00060647 |
|  | GO:0006508 | proteolysis | 0.00061428 |
|  | GO:0032103 | positive regulation of response to external stimulus | 0.0006729 |
|  | GO:0006807 | nitrogen compound metabolic process | 0.00080263 |
|  | GO:0031326 | regulation of cellular biosynthetic process | 0.00087202 |
|  | GO:0031667 | response to nutrient levels | 0.00096359 |
|  | GO:0031347 | regulation of defense response | 0.00099335 |
|  | GO:0048598 | embryonic morphogenesis | 0.00099844 |
|  | GO:0019221 | cytokine-mediated signaling pathway | 0.00100058 |
|  | GO:0009889 | regulation of biosynthetic process | 0.0010026 |
|  | GO:0002683 | negative regulation of immune system process | 0.00101954 |
|  | GO:0051252 | regulation of RNA metabolic process | 0.00113826 |
|  | GO:0034654 | nucleobase-containing compound biosynthetic process | 0.00113849 |
|  | GO:0010558 | negative regulation of macromolecule biosynthetic process | 0.00118163 |
|  | GO:0002252 | immune effector process | 0.00122748 |
|  | GO:0006366 | transcription by RNA polymerase II | 0.00131307 |
|  | GO:0016049 | cell growth | 0.00134433 |
|  | GO:0051241 | negative regulation of multicellular organismal process | 0.00137866 |
|  | GO:0009991 | response to extracellular stimulus | 0.00163701 |
|  | GO:0031327 | negative regulation of cellular biosynthetic process | 0.00164828 |
|  | GO:0018130 | heterocycle biosynthetic process | 0.00165268 |
|  | GO:0019438 | aromatic compound biosynthetic process | 0.00174676 |
|  | GO:0022008 | neurogenesis | 0.00204474 |
|  | GO:0010035 | response to inorganic substance | 0.0021993 |
|  | GO:0060284 | regulation of cell development | 0.00283238 |
|  | GO:0009792 | embryo development ending in birth or egg hatching | 0.0029586 |
|  | GO:1901362 | organic cyclic compound biosynthetic process | 0.00355692 |
|  | GO:0007417 | central nervous system development | 0.00427211 |
|  | GO:0007399 | nervous system development | 0.005644 |
|  | GO:0051050 | positive regulation of transport | 0.00715581 |
|  | GO:0051093 | negative regulation of developmental process | 0.00800419 |
|  | GO:0043170 | macromolecule metabolic process | 0.00842488 |
|  | GO:0051172 | negative regulation of nitrogen compound metabolic process | 0.00903395 |
|  | GO:0034660 | ncRNA metabolic process | 0.00907222 |
|  | GO:0051726 | regulation of cell cycle | 0.00941089 |
|  | GO:0007267 | cell-cell signaling | 0.00996008 |
|  | GO:0045087 | innate immune response | 0.01041329 |
|  | GO:0060322 | head development | 0.01177251 |
|  | GO:0007610 | behavior | 0.01393508 |
|  | GO:0044271 | cellular nitrogen compound biosynthetic process | 0.01439563 |
|  | GO:0048871 | multicellular organismal-level homeostasis | 0.01544064 |
|  | GO:0043009 | chordate embryonic development | 0.01656722 |
|  | GO:0061061 | muscle structure development | 0.01745932 |
|  | GO:0045596 | negative regulation of cell differentiation | 0.01912097 |
|  | GO:0098542 | defense response to other organism | 0.0207232 |
|  | GO:0044238 | primary metabolic process | 0.0239568 |
|  | GO:0008285 | negative regulation of cell population proliferation | 0.02702229 |
|  | GO:0016043 | cellular component organization | 0.0321404 |
|  | GO:0030855 | epithelial cell differentiation | 0.03467358 |
|  | GO:0032879 | regulation of localization | 0.0389728 |
|  | GO:0000278 | mitotic cell cycle | 0.04200493 |
|  | GO:0007420 | brain development | 0.0438355 |
|  | GO:0071704 | organic substance metabolic process | 0.04532164 |
| 2 | GO:0035556 | intracellular signal transduction | 1.02E-11 |
|  | GO:0042981 | regulation of apoptotic process | 2.35E-11 |
|  | GO:0043067 | regulation of programmed cell death | 3.74E-11 |
|  | GO:0033554 | cellular response to stress | 5.94E-11 |
|  | GO:0048522 | positive regulation of cellular process | 5.80E-10 |
|  | GO:0071214 | cellular response to abiotic stimulus | 5.88E-10 |
|  | GO:0104004 | cellular response to environmental stimulus | 5.88E-10 |
|  | GO:0042221 | response to chemical | 8.61E-10 |
|  | GO:0010033 | response to organic substance | 1.02E-09 |
|  | GO:0006915 | apoptotic process | 1.50E-09 |
|  | GO:0012501 | programmed cell death | 2.66E-09 |
|  | GO:0008219 | cell death | 2.79E-09 |
|  | GO:0048518 | positive regulation of biological process | 4.28E-09 |
|  | GO:0051173 | positive regulation of nitrogen compound metabolic process | 5.99E-09 |
|  | GO:0051247 | positive regulation of protein metabolic process | 7.96E-09 |
|  | GO:0009628 | response to abiotic stimulus | 1.18E-08 |
|  | GO:1901700 | response to oxygen-containing compound | 1.20E-08 |
|  | GO:0006950 | response to stress | 1.84E-08 |
|  | GO:0009967 | positive regulation of signal transduction | 2.85E-08 |
|  | GO:0043069 | negative regulation of programmed cell death | 2.86E-08 |
|  | GO:0014070 | response to organic cyclic compound | 2.97E-08 |
|  | GO:0023051 | regulation of signaling | 3.44E-08 |
|  | GO:0010646 | regulation of cell communication | 3.60E-08 |
|  | GO:0010604 | positive regulation of macromolecule metabolic process | 5.33E-08 |
|  | GO:0051128 | regulation of cellular component organization | 5.73E-08 |
|  | GO:1902533 | positive regulation of intracellular signal transduction | 6.31E-08 |
|  | GO:0009966 | regulation of signal transduction | 7.56E-08 |
|  | GO:0065009 | regulation of molecular function | 1.11E-07 |
|  | GO:1902531 | regulation of intracellular signal transduction | 1.31E-07 |
|  | GO:0009719 | response to endogenous stimulus | 1.36E-07 |
|  | GO:0023056 | positive regulation of signaling | 1.71E-07 |
|  | GO:0010647 | positive regulation of cell communication | 1.71E-07 |
|  | GO:0051716 | cellular response to stimulus | 1.74E-07 |
|  | GO:0009893 | positive regulation of metabolic process | 2.36E-07 |
|  | GO:0007507 | heart development | 2.40E-07 |
|  | GO:0043085 | positive regulation of catalytic activity | 2.41E-07 |
|  | GO:0050790 | regulation of catalytic activity | 2.76E-07 |
|  | GO:0070887 | cellular response to chemical stimulus | 2.92E-07 |
|  | GO:0080134 | regulation of response to stress | 2.96E-07 |
|  | GO:0072359 | circulatory system development | 3.23E-07 |
|  | GO:0097190 | apoptotic signaling pathway | 3.27E-07 |
|  | GO:0023052 | signaling | 3.62E-07 |
|  | GO:0071495 | cellular response to endogenous stimulus | 3.77E-07 |
|  | GO:0048583 | regulation of response to stimulus | 3.90E-07 |
|  | GO:0007166 | cell surface receptor signaling pathway | 4.87E-07 |
|  | GO:0007154 | cell communication | 4.97E-07 |
|  | GO:0043066 | negative regulation of apoptotic process | 5.10E-07 |
|  | GO:0080135 | regulation of cellular response to stress | 6.51E-07 |
|  | GO:0051246 | regulation of protein metabolic process | 8.58E-07 |
|  | GO:0071310 | cellular response to organic substance | 1.2344E-06 |
|  | GO:0050793 | regulation of developmental process | 1.338E-06 |
|  | GO:1901701 | cellular response to oxygen-containing compound | 1.3713E-06 |
|  | GO:0007165 | signal transduction | 2.048E-06 |
|  | GO:0048513 | animal organ development | 2.1288E-06 |
|  | GO:0000165 | MAPK cascade | 2.8358E-06 |
|  | GO:0042127 | regulation of cell population proliferation | 3.9278E-06 |
|  | GO:0048584 | positive regulation of response to stimulus | 4.6458E-06 |
|  | GO:0019222 | regulation of metabolic process | 5.4229E-06 |
|  | GO:0019538 | protein metabolic process | 6.1078E-06 |
|  | GO:0044093 | positive regulation of molecular function | 9.2348E-06 |
|  | GO:0031325 | positive regulation of cellular metabolic process | 1.0279E-05 |
|  | GO:0050896 | response to stimulus | 1.1681E-05 |
|  | GO:0033993 | response to lipid | 1.4374E-05 |
|  | GO:0051239 | regulation of multicellular organismal process | 1.8279E-05 |
|  | GO:0008284 | positive regulation of cell population proliferation | 1.9917E-05 |
|  | GO:0007167 | enzyme-linked receptor protein signaling pathway | 2.4718E-05 |
|  | GO:0060341 | regulation of cellular localization | 3.1269E-05 |
|  | GO:0010243 | response to organonitrogen compound | 4.8711E-05 |
|  | GO:0009653 | anatomical structure morphogenesis | 5.7901E-05 |
|  | GO:0031328 | positive regulation of cellular biosynthetic process | 5.8181E-05 |
|  | GO:0009605 | response to external stimulus | 5.8745E-05 |
|  | GO:0009891 | positive regulation of biosynthetic process | 6.3747E-05 |
|  | GO:0035295 | tube development | 7.8307E-05 |
|  | GO:0048731 | system development | 9.2834E-05 |
|  | GO:1901698 | response to nitrogen compound | 9.387E-05 |
|  | GO:0051130 | positive regulation of cellular component organization | 9.8793E-05 |
|  | GO:0051726 | regulation of cell cycle | 9.9635E-05 |
|  | GO:1901564 | organonitrogen compound metabolic process | 0.00012678 |
|  | GO:0006468 | protein phosphorylation | 0.00012724 |
|  | GO:0051171 | regulation of nitrogen compound metabolic process | 0.00016627 |
|  | GO:0010628 | positive regulation of gene expression | 0.00017845 |
|  | GO:0080090 | regulation of primary metabolic process | 0.00028116 |
|  | GO:0051240 | positive regulation of multicellular organismal process | 0.00031058 |
|  | GO:0031323 | regulation of cellular metabolic process | 0.00034074 |
|  | GO:0048870 | cell motility | 0.00041493 |
|  | GO:0048519 | negative regulation of biological process | 0.00042704 |
|  | GO:0010557 | positive regulation of macromolecule biosynthetic process | 0.00043119 |
|  | GO:0051094 | positive regulation of developmental process | 0.00046332 |
|  | GO:0016310 | phosphorylation | 0.00058958 |
|  | GO:0008283 | cell population proliferation | 0.00059723 |
|  | GO:0007275 | multicellular organism development | 0.00088471 |
|  | GO:0006810 | transport | 0.00122878 |
|  | GO:0036211 | protein modification process | 0.00123897 |
|  | GO:0048523 | negative regulation of cellular process | 0.00137736 |
|  | GO:0016477 | cell migration | 0.00139127 |
|  | GO:0051234 | establishment of localization | 0.0021627 |
|  | GO:0045595 | regulation of cell differentiation | 0.00235608 |
|  | GO:0043412 | macromolecule modification | 0.00270734 |
|  | GO:0051641 | cellular localization | 0.0029398 |
|  | GO:0030154 | cell differentiation | 0.00302494 |
|  | GO:0048869 | cellular developmental process | 0.00303433 |
|  | GO:0060255 | regulation of macromolecule metabolic process | 0.00396678 |
|  | GO:0032879 | regulation of localization | 0.00446212 |
|  | GO:0050794 | regulation of cellular process | 0.00469949 |
|  | GO:0044419 | biological process involved in interspecies interaction between organisms | 0.00504681 |
|  | GO:0008104 | protein localization | 0.00682817 |
|  | GO:0051049 | regulation of transport | 0.00705741 |
|  | GO:0070727 | cellular macromolecule localization | 0.00710006 |
|  | GO:0006508 | proteolysis | 0.00720361 |
|  | GO:0048468 | cell development | 0.00779242 |
|  | GO:0007049 | cell cycle | 0.00904062 |
|  | GO:0051179 | localization | 0.0122538 |
|  | GO:0050789 | regulation of biological process | 0.01339906 |
|  | GO:0032502 | developmental process | 0.01384031 |
|  | GO:0009888 | tissue development | 0.02238894 |
|  | GO:0045935 | positive regulation of nucleobase-containing compound metabolic process | 0.02611159 |
|  | GO:0048856 | anatomical structure development | 0.02685968 |
|  | GO:0065007 | biological regulation | 0.0280126 |
|  | GO:0032501 | multicellular organismal process | 0.02814697 |
|  | GO:0009056 | catabolic process | 0.03433119 |
|  | GO:0033036 | macromolecule localization | 0.03720604 |
|  | GO:0006796 | phosphate-containing compound metabolic process | 0.04551179 |
|  | GO:0006793 | phosphorus metabolic process | 0.04971538 |
| 4 | GO:0010628 | positive regulation of gene expression | 2.04E-08 |
|  | GO:0042981 | regulation of apoptotic process | 2.43E-07 |
|  | GO:0043067 | regulation of programmed cell death | 3.44E-07 |
|  | GO:0051173 | positive regulation of nitrogen compound metabolic process | 5.2848E-06 |
|  | GO:0006915 | apoptotic process | 5.3135E-06 |
|  | GO:0012501 | programmed cell death | 8.1243E-06 |
|  | GO:0008219 | cell death | 8.4107E-06 |
|  | GO:0010557 | positive regulation of macromolecule biosynthetic process | 1.1294E-05 |
|  | GO:0031328 | positive regulation of cellular biosynthetic process | 1.6816E-05 |
|  | GO:0070887 | cellular response to chemical stimulus | 1.7366E-05 |
|  | GO:0009891 | positive regulation of biosynthetic process | 1.8348E-05 |
|  | GO:0008284 | positive regulation of cell population proliferation | 2.3276E-05 |
|  | GO:0010033 | response to organic substance | 2.538E-05 |
|  | GO:0010604 | positive regulation of macromolecule metabolic process | 2.8371E-05 |
|  | GO:0051171 | regulation of nitrogen compound metabolic process | 8.2845E-05 |
|  | GO:0009893 | positive regulation of metabolic process | 8.9087E-05 |
|  | GO:0071495 | cellular response to endogenous stimulus | 0.00010261 |
|  | GO:0080090 | regulation of primary metabolic process | 0.00013439 |
|  | GO:0071310 | cellular response to organic substance | 0.00015034 |
|  | GO:0042221 | response to chemical | 0.00016304 |
|  | GO:0051240 | positive regulation of multicellular organismal process | 0.00029297 |
|  | GO:0031325 | positive regulation of cellular metabolic process | 0.00031274 |
|  | GO:0042127 | regulation of cell population proliferation | 0.00037114 |
|  | GO:0009719 | response to endogenous stimulus | 0.0004281 |
|  | GO:0045893 | positive regulation of DNA-templated transcription | 0.00043527 |
|  | GO:1902680 | positive regulation of RNA biosynthetic process | 0.00045241 |
|  | GO:0030154 | cell differentiation | 0.00047147 |
|  | GO:0048869 | cellular developmental process | 0.0004729 |
|  | GO:1901700 | response to oxygen-containing compound | 0.00065271 |
|  | GO:0051246 | regulation of protein metabolic process | 0.00093118 |
|  | GO:0051254 | positive regulation of RNA metabolic process | 0.00094201 |
|  | GO:0048513 | animal organ development | 0.00112887 |
|  | GO:0007275 | multicellular organism development | 0.00113351 |
|  | GO:0060255 | regulation of macromolecule metabolic process | 0.00155898 |
|  | GO:0065009 | regulation of molecular function | 0.00164334 |
|  | GO:0048522 | positive regulation of cellular process | 0.00170677 |
|  | GO:0008283 | cell population proliferation | 0.00191441 |
|  | GO:0045935 | positive regulation of nucleobase-containing compound metabolic process | 0.00238572 |
|  | GO:0048856 | anatomical structure development | 0.00245807 |
|  | GO:0009653 | anatomical structure morphogenesis | 0.00302686 |
|  | GO:0023051 | regulation of signaling | 0.00346244 |
|  | GO:0019222 | regulation of metabolic process | 0.0047938 |
|  | GO:0048518 | positive regulation of biological process | 0.00603517 |
|  | GO:0051239 | regulation of multicellular organismal process | 0.00834259 |
|  | GO:0009966 | regulation of signal transduction | 0.00848397 |
|  | GO:0032502 | developmental process | 0.00858002 |
|  | GO:0051128 | regulation of cellular component organization | 0.01144274 |
|  | GO:0050793 | regulation of developmental process | 0.0135775 |
|  | GO:0006950 | response to stress | 0.01600255 |
|  | GO:0048583 | regulation of response to stimulus | 0.01600255 |
|  | GO:0048731 | system development | 0.01795545 |
|  | GO:0035556 | intracellular signal transduction | 0.02268366 |
|  | GO:0048519 | negative regulation of biological process | 0.02749381 |
|  | GO:0010646 | regulation of cell communication | 0.0324892 |
|  | GO:0006355 | regulation of DNA-templated transcription | 0.03560791 |
|  | GO:0048468 | cell development | 0.03782957 |
|  | GO:2001141 | regulation of RNA biosynthetic process | 0.03800987 |
|  | GO:0007166 | cell surface receptor signaling pathway | 0.04047866 |
|  | GO:0050794 | regulation of cellular process | 0.04504668 |

RCCC, Renal clear cell carcinoma.

**Supplementary Data 6.** Molecular functions linked to RCCC.

| **Term ID** | **Term name** | **FDR** |
| --- | --- | --- |
| GO:0005515 | protein binding | 4.78E-22 |
| GO:0019899 | enzyme binding | 2.39E-18 |
| GO:0005102 | signaling receptor binding | 1.45E-17 |
| GO:0042802 | identical protein binding | 5.30E-13 |
| GO:0044877 | protein-containing complex binding | 6.17E-12 |
| GO:0019901 | protein kinase binding | 1.04E-09 |
| GO:0043167 | ion binding | 1.71E-09 |
| GO:0019900 | kinase binding | 4.57E-09 |
| GO:0019904 | protein domain specific binding | 2.97E-08 |
| GO:0140677 | molecular function activator activity | 6.99E-08 |
| GO:0098772 | molecular function regulator activity | 7.18E-08 |
| GO:0140096 | catalytic activity, acting on a protein | 1.38E-07 |
| GO:0050839 | cell adhesion molecule binding | 1.077E-06 |
| GO:0004672 | protein kinase activity | 3.4143E-06 |
| GO:0008270 | zinc ion binding | 7.2076E-06 |
| GO:0042277 | peptide binding | 8.6885E-06 |
| GO:0005126 | cytokine receptor binding | 9.1734E-06 |
| GO:0036094 | small molecule binding | 1.1864E-05 |
| GO:0046983 | protein dimerization activity | 1.4844E-05 |
| GO:0097367 | carbohydrate derivative binding | 1.489E-05 |
| GO:0043168 | anion binding | 1.5575E-05 |
| GO:0046914 | transition metal ion binding | 1.6696E-05 |
| GO:0016773 | phosphotransferase activity, alcohol group as acceptor | 2.8996E-05 |
| GO:0005488 | binding | 3.534E-05 |
| GO:0016301 | kinase activity | 3.7975E-05 |
| GO:0008134 | transcription factor binding | 5.543E-05 |
| GO:0031625 | ubiquitin protein ligase binding | 6.5661E-05 |
| GO:0042803 | protein homodimerization activity | 6.7188E-05 |
| GO:0030546 | signaling receptor activator activity | 8.204E-05 |
| GO:0043565 | sequence-specific DNA binding | 9.0342E-05 |
| GO:0001228 | DNA-binding transcription activator activity, RNA polymerase II-specific | 0.0001067 |
| GO:0030545 | signaling receptor regulator activity | 0.00011391 |
| GO:0001216 | DNA-binding transcription activator activity | 0.00012744 |
| GO:0048018 | receptor ligand activity | 0.00018188 |
| GO:0044389 | ubiquitin-like protein ligase binding | 0.00018249 |
| GO:0033218 | amide binding | 0.00019245 |
| GO:0004674 | protein serine/threonine kinase activity | 0.00023217 |
| GO:0046872 | metal ion binding | 0.00031529 |
| GO:0043169 | cation binding | 0.00033454 |
| GO:0003682 | chromatin binding | 0.00036491 |
| GO:0016740 | transferase activity | 0.00038128 |
| GO:0005178 | integrin binding | 0.00047612 |
| GO:0001221 | transcription coregulator binding | 0.00048781 |
| GO:0019207 | kinase regulator activity | 0.00049755 |
| GO:0070851 | growth factor receptor binding | 0.00074651 |
| GO:1990837 | sequence-specific double-stranded DNA binding | 0.000784 |
| GO:0016772 | transferase activity, transferring phosphorus-containing groups | 0.0012421 |
| GO:0140110 | transcription regulator activity | 0.00124343 |
| GO:0003690 | double-stranded DNA binding | 0.0013059 |
| GO:0003824 | catalytic activity | 0.00153846 |
| GO:0000976 | transcription cis-regulatory region binding | 0.00155125 |
| GO:0001067 | transcription regulatory region nucleic acid binding | 0.00161947 |
| GO:0030554 | adenyl nucleotide binding | 0.00206905 |
| GO:0140297 | DNA-binding transcription factor binding | 0.00210739 |
| GO:0017076 | purine nucleotide binding | 0.00321915 |
| GO:0019887 | protein kinase regulator activity | 0.00354588 |
| GO:0008083 | growth factor activity | 0.00419533 |
| GO:0032559 | adenyl ribonucleotide binding | 0.00491047 |
| GO:0032553 | ribonucleotide binding | 0.00521236 |
| GO:0005125 | cytokine activity | 0.0055859 |
| GO:0005524 | ATP binding | 0.00621833 |
| GO:0000166 | nucleotide binding | 0.00705995 |
| GO:1901265 | nucleoside phosphate binding | 0.00717273 |
| GO:0032555 | purine ribonucleotide binding | 0.00745483 |
| GO:0061629 | RNA polymerase II-specific DNA-binding transcription factor binding | 0.00859656 |
| GO:0035639 | purine ribonucleoside triphosphate binding | 0.00869272 |
| GO:0003677 | DNA binding | 0.01162024 |
| GO:0106310 | protein serine kinase activity | 0.01253729 |
| GO:0019838 | growth factor binding | 0.01319954 |
| GO:0019902 | phosphatase binding | 0.02530963 |
| GO:0019209 | kinase activator activity | 0.0269809 |
| GO:0000981 | DNA-binding transcription factor activity, RNA polymerase II-specific | 0.03310608 |
| GO:0045296 | cadherin binding | 0.04476297 |
| GO:0030295 | protein kinase activator activity | 0.04518392 |

RCCC, Renal clear cell carcinoma.

**Supplementary Data 7.** Cellular components linked to RCCC.

| **Term ID** | **Term name** | **FDR** |
| --- | --- | --- |
| GO:0005737 | cytoplasm | 5.49E-17 |
| GO:0071944 | cell periphery | 1.71E-13 |
| GO:0005886 | plasma membrane | 5.62E-13 |
| GO:0009986 | cell surface | 1.14E-12 |
| GO:0031982 | vesicle | 1.44E-12 |
| GO:0030054 | cell junction | 2.87E-11 |
| GO:0098857 | membrane microdomain | 6.87E-11 |
| GO:0031410 | cytoplasmic vesicle | 7.59E-11 |
| GO:0005654 | nucleoplasm | 8.19E-11 |
| GO:0097708 | intracellular vesicle | 8.59E-11 |
| GO:0012505 | endomembrane system | 1.81E-10 |
| GO:0005615 | extracellular space | 2.98E-10 |
| GO:0045121 | membrane raft | 3.93E-10 |
| GO:0016020 | membrane | 1.83E-09 |
| GO:0005576 | extracellular region | 3.17E-09 |
| GO:0043233 | organelle lumen | 2.00E-08 |
| GO:0031974 | membrane-enclosed lumen | 2.00E-08 |
| GO:0070013 | intracellular organelle lumen | 2.00E-08 |
| GO:0048471 | perinuclear region of cytoplasm | 6.14E-08 |
| GO:0070161 | anchoring junction | 1.17E-07 |
| GO:0005829 | cytosol | 2.08E-07 |
| GO:0043227 | membrane-bounded organelle | 1.32088E-06 |
| GO:0005768 | endosome | 1.63625E-06 |
| GO:0098588 | bounding membrane of organelle | 2.27615E-06 |
| GO:0043231 | intracellular membrane-bounded organelle | 3.95159E-06 |
| GO:0098552 | side of membrane | 4.04599E-06 |
| GO:0045202 | synapse | 6.91289E-06 |
| GO:0012506 | vesicle membrane | 1.54063E-05 |
| GO:0031981 | nuclear lumen | 1.95692E-05 |
| GO:0030659 | cytoplasmic vesicle membrane | 6.22155E-05 |
| GO:0044853 | plasma membrane raft | 7.53915E-05 |
| GO:0043226 | organelle | 9.52696E-05 |
| GO:0099503 | secretory vesicle | 0.000174826 |
| GO:0031252 | cell leading edge | 0.000200168 |
| GO:0030141 | secretory granule | 0.000207194 |
| GO:0098590 | plasma membrane region | 0.000227044 |
| GO:0009897 | external side of plasma membrane | 0.000227857 |
| GO:0019898 | extrinsic component of membrane | 0.000228362 |
| GO:0042995 | cell projection | 0.000235188 |
| GO:0005667 | transcription regulator complex | 0.000261187 |
| GO:0030666 | endocytic vesicle membrane | 0.000314433 |
| GO:1902911 | protein kinase complex | 0.000554209 |
| GO:0120025 | plasma membrane bounded cell projection | 0.000566132 |
| GO:0070062 | extracellular exosome | 0.000729615 |
| GO:0005911 | cell-cell junction | 0.000735614 |
| GO:0098797 | plasma membrane protein complex | 0.000827268 |
| GO:1903561 | extracellular vesicle | 0.00108543 |
| GO:0043230 | extracellular organelle | 0.001103315 |
| GO:0065010 | extracellular membrane-bounded organelle | 0.001103315 |
| GO:0005901 | caveola | 0.001110924 |
| GO:1902494 | catalytic complex | 0.001377086 |
| GO:0098796 | membrane protein complex | 0.001598682 |
| GO:0032993 | protein-DNA complex | 0.001628053 |
| GO:0036477 | somatodendritic compartment | 0.001949271 |
| GO:0005925 | focal adhesion | 0.002019832 |
| GO:0150034 | distal axon | 0.002044969 |
| GO:0045334 | clathrin-coated endocytic vesicle | 0.002119782 |
| GO:0098978 | glutamatergic synapse | 0.002448621 |
| GO:0005769 | early endosome | 0.002642144 |
| GO:0030055 | cell-substrate junction | 0.002958553 |
| GO:0001726 | ruffle | 0.003156927 |
| GO:0031090 | organelle membrane | 0.003342774 |
| GO:0044297 | cell body | 0.003985042 |
| GO:0000785 | chromatin | 0.004104642 |
| GO:0030424 | axon | 0.004959349 |
| GO:0061695 | transferase complex, transferring phosphorus-containing groups | 0.005115167 |
| GO:1990234 | transferase complex | 0.005692471 |
| GO:0032587 | ruffle membrane | 0.006650626 |
| GO:0005770 | late endosome | 0.007541877 |
| GO:0005694 | chromosome | 0.009967832 |
| GO:0030139 | endocytic vesicle | 0.010337422 |
| GO:0043229 | intracellular organelle | 0.010467047 |
| GO:0005938 | cell cortex | 0.011071418 |
| GO:0090575 | RNA polymerase II transcription regulator complex | 0.01181575 |
| GO:0019897 | extrinsic component of plasma membrane | 0.012498415 |
| GO:0005622 | intracellular anatomical structure | 0.013985904 |
| GO:0045178 | basal part of cell | 0.017824758 |
| GO:1902554 | serine/threonine protein kinase complex | 0.018932152 |
| GO:0005794 | Golgi apparatus | 0.019243185 |
| GO:0005773 | vacuole | 0.02073494 |
| GO:0005765 | lysosomal membrane | 0.021401713 |
| GO:0098852 | lytic vacuole membrane | 0.021401713 |
| GO:0016323 | basolateral plasma membrane | 0.022786518 |
| GO:0031968 | organelle outer membrane | 0.028623624 |
| GO:0043025 | neuronal cell body | 0.029757925 |
| GO:0005764 | lysosome | 0.030296333 |
| GO:0000323 | lytic vacuole | 0.030296333 |
| GO:0005774 | vacuolar membrane | 0.030918376 |
| GO:0019867 | outer membrane | 0.031301196 |
| GO:0043005 | neuron projection | 0.043923665 |
| GO:0099572 | postsynaptic specialization | 0.047630606 |

RCCC, Renal clear cell carcinoma.


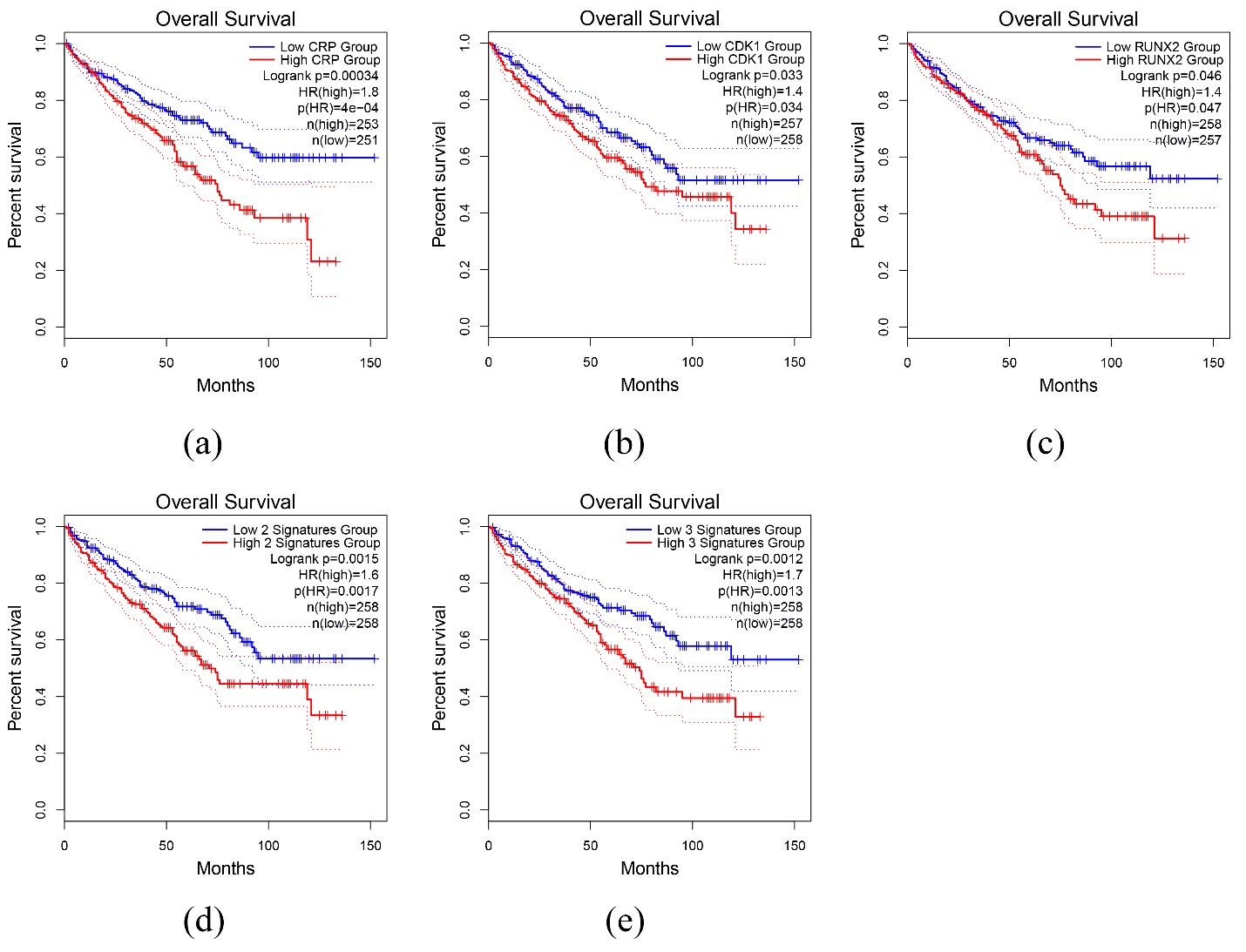
**Supplementary Data 8.** Kaplan-Meier survival curves were generated for various biomarkers, namely (a) CRP, (b) CDK1, (c) RUNX2, (d) CRP + CDK1, and (e) CRP + CDK1 + RUNX2, in patients diagnosed with RCCC. The curves depict distinct outcomes based on the expression levels of these markers, with blue and red lines representing under and over-expression, respectively. The y-axis illustrates the probability of survival, while the x-axis delineates the survival months for RCCC patients. Additionally, 95% confidence intervals are depicted by dotted lines, providing a comprehensive visual representation of the survival analysis. RCCC, Renal clear cell carcinoma.


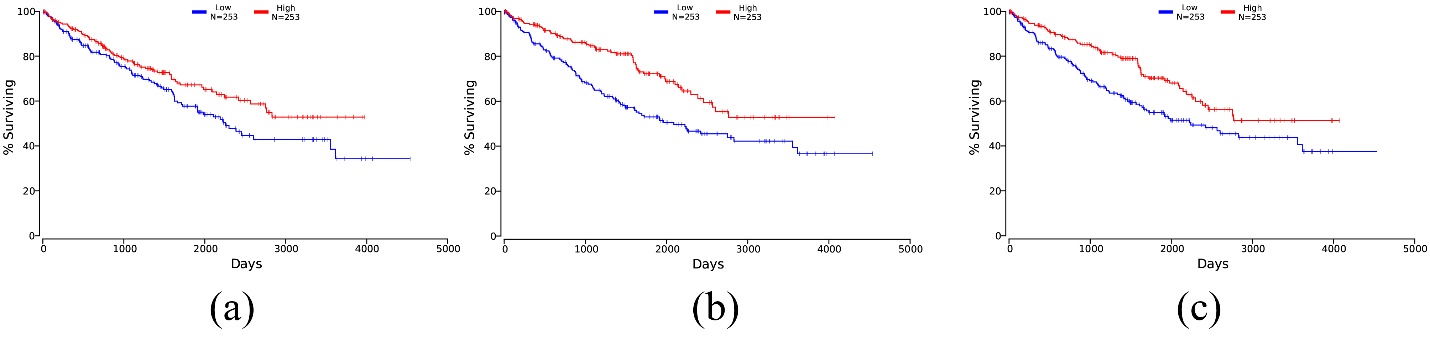


**Supplementary Data 9.** Prognostic role of (a) has-miR-26a-1-3p, (b) has-miR-144-3P, and (c) has-miR-144-5P I patients with renal clear cell carcinoma.


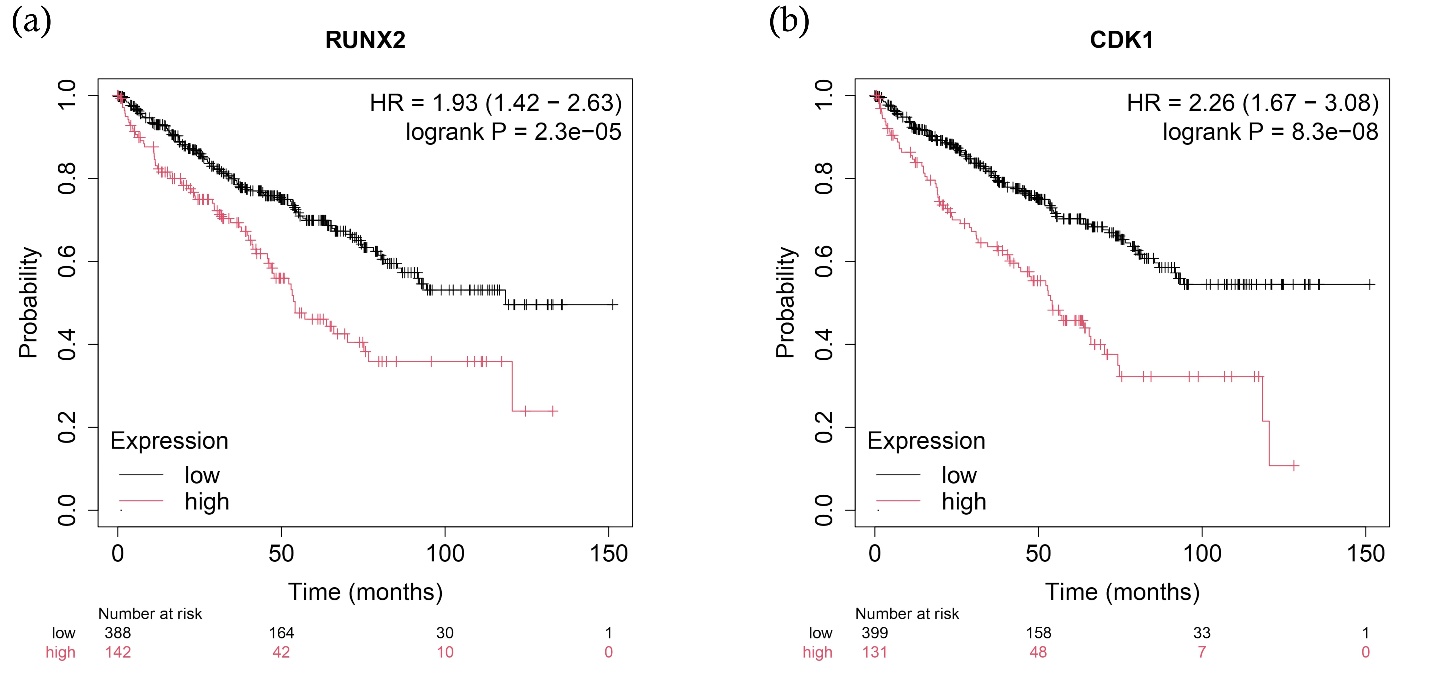


**Supplementary Data 10.** Overall survival analysis of **(a)** RUNX2 and **(b)** CDK1 in KIRC patients was generated using RNA-Seq data with the Kaplan-Meier plotter tool. Patients were stratified into "high" and "low" expression groups based on the median expression level of each gene. High expression of both RUNX2 (HR = 1.93, log-rank p = 2.3e-05) and CDK1 (HR = 2.26, log-rank p = 8.3e-08) is significantly correlated with reduced overall survival, confirming their role as strong negative prognostic biomarkers in KIRC. KIRC, Kidney renal clear cell carcinoma.

**
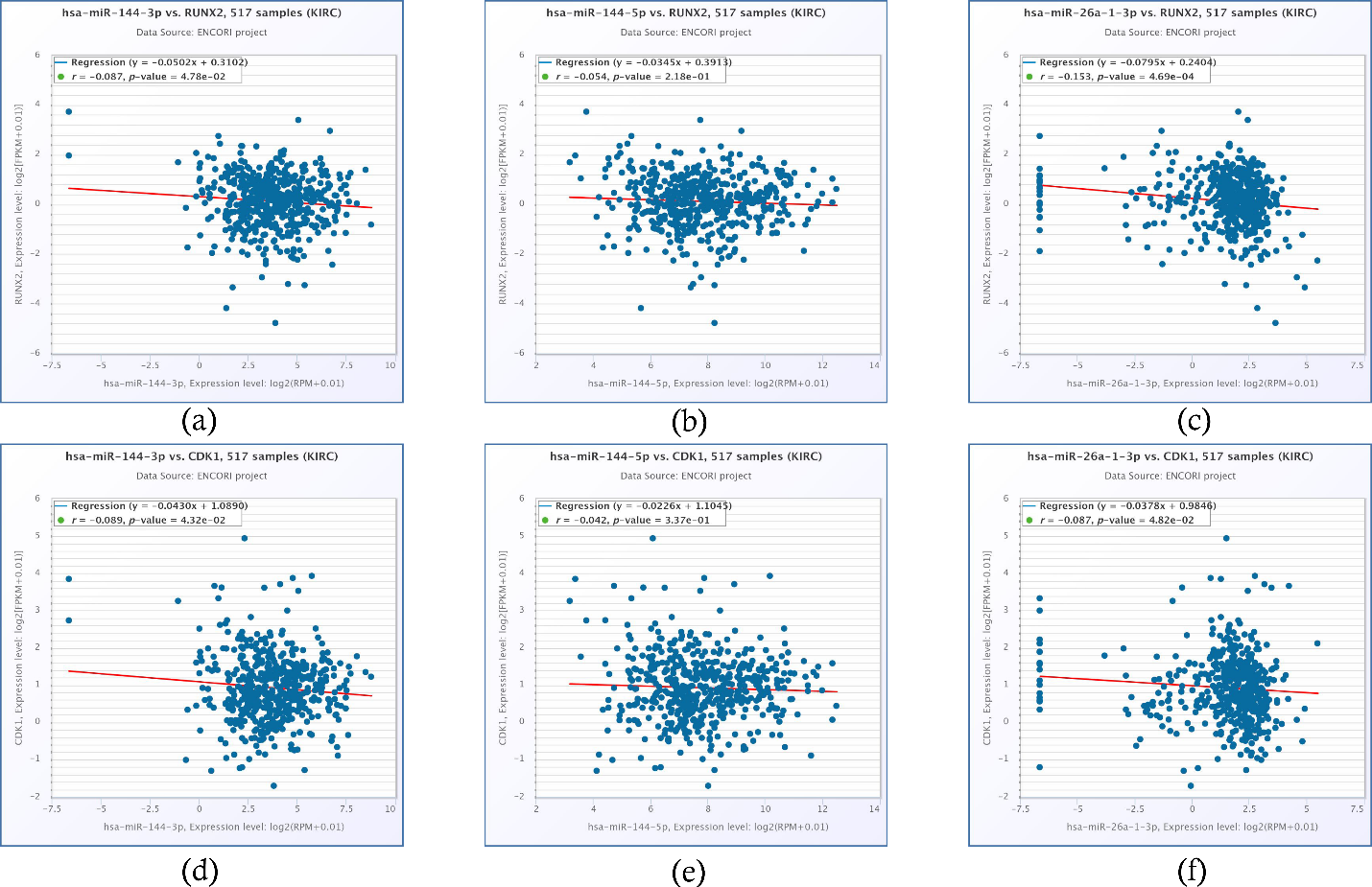
**

**Supplementary Data 11.**

Scatter plots depicting the correlation between the expression levels of key miRNAs and hub genes in 517 RCCC samples from the starBase database. The analysis shows the relationship for: (a) hsa-miR-144-3p vs. RUNX2, (b) hsa-miR-144-5p vs. RUNX2, (c) hsa-miR-26a-1-3p vs. RUNX2, (d) hsa-miR-144-3p vs. CDK1, (e) hsa-miR-144-5p vs. CDK1, and (f) hsa-miR-26a-1-3p vs. CDK1. KIRC, Kidney renal clear cell carcinoma.
